# Disruption of chromatin folding domains by somatic genomic rearrangements in human cancer

**DOI:** 10.1038/s41588-019-0564-y

**Published:** 2020-02-05

**Authors:** Kadir C. Akdemir, Victoria T. Le, Sahaana Chandran, Yilong Li, Roel G. Verhaak, Rameen Beroukhim, Peter J. Campbell, Lynda Chin, Jesse R. Dixon, P. Andrew Futreal, PCAWG Structural Variation Working Group, Kadir C. Akdemir, Eva G. Alvarez, Adrian Baez-Ortega, Paul C. Boutros, David D. L. Bowtell, Benedikt Brors, Kathleen H. Burns, Peter J. Campbell, Kin Chan, Ken Chen, Isidro Cortés-Ciriano, Ana Dueso-Barroso, Andrew J. Dunford, Paul A. Edwards, Xavier Estivill, Dariush Etemadmoghadam, Lars Feuerbach, J. Lynn Fink, Milana Frenkel-Morgenstern, Dale W. Garsed, Mark Gerstein, Dmitry A. Gordenin, David Haan, James E. Haber, Julian M. Hess, Barbara Hutter, Marcin Imielinski, David T. W. Jones, Young Seok Ju, Marat D. Kazanov, Leszek J. Klimczak, Youngil Koh, Jan O. Korbel, Kiran Kumar, Eunjung Alice Lee, Jake June-Koo Lee, Yilong Li, Andy G. Lynch, Geoff Macintyre, Florian Markowetz, Iñigo Martincorena, Alexander Martinez-Fundichely, Matthew Meyerson, Satoru Miyano, Hidewaki Nakagawa, Fabio C. P. Navarro, Stephan Ossowski, Peter J. Park, John V. Pearson, Montserrat Puiggròs, Karsten Rippe, Nicola D. Roberts, Steven A. Roberts, Bernardo Rodriguez-Martin, Steven E. Schumacher, Ralph Scully, Mark Shackleton, Nikos Sidiropoulos, Lina Sieverling, Chip Stewart, David Torrents, Jose M. C. Tubio, Izar Villasante, Nicola Waddell, Jeremiah A. Wala, Joachim Weischenfeldt, Lixing Yang, Xiaotong Yao, Sung-Soo Yoon, Jorge Zamora, Cheng-Zhong Zhang, PCAWG Consortium, Lauri A. Aaltonen, Federico Abascal, Adam Abeshouse, Hiroyuki Aburatani, David J. Adams, Nishant Agrawal, Keun Soo Ahn, Sung-Min Ahn, Hiroshi Aikata, Rehan Akbani, Kadir C. Akdemir, Hikmat Al-Ahmadie, Sultan T. Al-Sedairy, Fatima Al-Shahrour, Malik Alawi, Monique Albert, Kenneth Aldape, Ludmil B. Alexandrov, Adrian Ally, Kathryn Alsop, Eva G. Alvarez, Fernanda Amary, Samirkumar B. Amin, Brice Aminou, Ole Ammerpohl, Matthew J. Anderson, Yeng Ang, Davide Antonello, Pavana Anur, Samuel Aparicio, Elizabeth L. Appelbaum, Yasuhito Arai, Axel Aretz, Koji Arihiro, Shun-ichi Ariizumi, Joshua Armenia, Laurent Arnould, Sylvia Asa, Yassen Assenov, Gurnit Atwal, Sietse Aukema, J. Todd Auman, Miriam R. R. Aure, Philip Awadalla, Marta Aymerich, Gary D. Bader, Adrian Baez-Ortega, Matthew H. Bailey, Peter J. Bailey, Miruna Balasundaram, Saianand Balu, Pratiti Bandopadhayay, Rosamonde E. Banks, Stefano Barbi, Andrew P. Barbour, Jonathan Barenboim, Jill Barnholtz-Sloan, Hugh Barr, Elisabet Barrera, John Bartlett, Javier Bartolome, Claudio Bassi, Oliver F. Bathe, Daniel Baumhoer, Prashant Bavi, Stephen B. Baylin, Wojciech Bazant, Duncan Beardsmore, Timothy A. Beck, Sam Behjati, Andreas Behren, Beifang Niu, Cindy Bell, Sergi Beltran, Christopher Benz, Andrew Berchuck, Anke K. Bergmann, Erik N. Bergstrom, Benjamin P. Berman, Daniel M. Berney, Stephan H. Bernhart, Rameen Beroukhim, Mario Berrios, Samantha Bersani, Johanna Bertl, Miguel Betancourt, Vinayak Bhandari, Shriram G. Bhosle, Andrew V. Biankin, Matthias Bieg, Darell Bigner, Hans Binder, Ewan Birney, Michael Birrer, Nidhan K. Biswas, Bodil Bjerkehagen, Tom Bodenheimer, Lori Boice, Giada Bonizzato, Johann S. De Bono, Arnoud Boot, Moiz S. Bootwalla, Ake Borg, Arndt Borkhardt, Keith A. Boroevich, Ivan Borozan, Christoph Borst, Marcus Bosenberg, Mattia Bosio, Jacqueline Boultwood, Guillaume Bourque, Paul C. Boutros, G. Steven Bova, David T. Bowen, Reanne Bowlby, David D. L. Bowtell, Sandrine Boyault, Rich Boyce, Jeffrey Boyd, Alvis Brazma, Paul Brennan, Daniel S. Brewer, Arie B. Brinkman, Robert G. Bristow, Russell R. Broaddus, Jane E. Brock, Malcolm Brock, Annegien Broeks, Angela N. Brooks, Denise Brooks, Benedikt Brors, Søren Brunak, Timothy J. C. Bruxner, Alicia L. Bruzos, Alex Buchanan, Ivo Buchhalter, Christiane Buchholz, Susan Bullman, Hazel Burke, Birgit Burkhardt, Kathleen H. Burns, John Busanovich, Carlos D. Bustamante, Adam P. Butler, Atul J. Butte, Niall J. Byrne, Anne-Lise Børresen-Dale, Samantha J. Caesar-Johnson, Andy Cafferkey, Declan Cahill, Claudia Calabrese, Carlos Caldas, Fabien Calvo, Niedzica Camacho, Peter J. Campbell, Elias Campo, Cinzia Cantù, Shaolong Cao, Thomas E. Carey, Joana Carlevaro-Fita, Rebecca Carlsen, Ivana Cataldo, Mario Cazzola, Jonathan Cebon, Robert Cerfolio, Dianne E. Chadwick, Dimple Chakravarty, Don Chalmers, Calvin Wing Yiu Chan, Kin Chan, Michelle Chan-Seng-Yue, Vishal S. Chandan, David K. Chang, Stephen J. Chanock, Lorraine A. Chantrill, Aurélien Chateigner, Nilanjan Chatterjee, Kazuaki Chayama, Hsiao-Wei Chen, Jieming Chen, Ken Chen, Yiwen Chen, Zhaohong Chen, Andrew D. Cherniack, Jeremy Chien, Yoke-Eng Chiew, Suet-Feung Chin, Juok Cho, Sunghoon Cho, Jung Kyoon Choi, Wan Choi, Christine Chomienne, Zechen Chong, Su Pin Choo, Angela Chou, Angelika N. Christ, Elizabeth L. Christie, Eric Chuah, Carrie Cibulskis, Kristian Cibulskis, Sara Cingarlini, Peter Clapham, Alexander Claviez, Sean Cleary, Nicole Cloonan, Marek Cmero, Colin C. Collins, Ashton A. Connor, Susanna L. Cooke, Colin S. Cooper, Leslie Cope, Vincenzo Corbo, Matthew G. Cordes, Stephen M. Cordner, Isidro Cortés-Ciriano, Kyle Covington, Prue A. Cowin, Brian Craft, David Craft, Chad J. Creighton, Yupeng Cun, Erin Curley, Ioana Cutcutache, Karolina Czajka, Bogdan Czerniak, Rebecca A. Dagg, Ludmila Danilova, Maria Vittoria Davi, Natalie R. Davidson, Helen Davies, Ian J. Davis, Brandi N. Davis-Dusenbery, Kevin J. Dawson, Francisco M. De La Vega, Ricardo De Paoli-Iseppi, Timothy Defreitas, Angelo P. Dei Tos, Olivier Delaneau, John A. Demchok, Jonas Demeulemeester, German M. Demidov, Deniz Demircioğlu, Nening M. Dennis, Robert E. Denroche, Stefan C. Dentro, Nikita Desai, Vikram Deshpande, Amit G. Deshwar, Christine Desmedt, Jordi Deu-Pons, Noreen Dhalla, Neesha C. Dhani, Priyanka Dhingra, Rajiv Dhir, Anthony DiBiase, Klev Diamanti, Li Ding, Shuai Ding, Huy Q. Dinh, Luc Dirix, HarshaVardhan Doddapaneni, Nilgun Donmez, Michelle T. Dow, Ronny Drapkin, Oliver Drechsel, Ruben M. Drews, Serge Serge, Tim Dudderidge, Ana Dueso-Barroso, Andrew J. Dunford, Michael Dunn, Lewis Jonathan Dursi, Fraser R. Duthie, Ken Dutton-Regester, Jenna Eagles, Douglas F. Easton, Stuart Edmonds, Paul A. Edwards, Sandra E. Edwards, Rosalind A. Eeles, Anna Ehinger, Juergen Eils, Roland Eils, Adel El-Naggar, Matthew Eldridge, Kyle Ellrott, Serap Erkek, Georgia Escaramis, Shadrielle M. G. Espiritu, Xavier Estivill, Dariush Etemadmoghadam, Jorunn E. Eyfjord, Bishoy M. Faltas, Daiming Fan, Yu Fan, William C. Faquin, Claudiu Farcas, Matteo Fassan, Aquila Fatima, Francesco Favero, Nodirjon Fayzullaev, Ina Felau, Sian Fereday, Martin L. Ferguson, Vincent Ferretti, Lars Feuerbach, Matthew A. Field, J. Lynn Fink, Gaetano Finocchiaro, Cyril Fisher, Matthew W. Fittall, Anna Fitzgerald, Rebecca C. Fitzgerald, Adrienne M. Flanagan, Neil E. Fleshner, Paul Flicek, John A. Foekens, Kwun M. Fong, Nuno A. Fonseca, Christopher S. Foster, Natalie S. Fox, Michael Fraser, Scott Frazer, Milana Frenkel-Morgenstern, William Friedman, Joan Frigola, Catrina C. Fronick, Akihiro Fujimoto, Masashi Fujita, Masashi Fukayama, Lucinda A. Fulton, Robert S. Fulton, Mayuko Furuta, P. Andrew Futreal, Anja Füllgrabe, Stacey B. Gabriel, Steven Gallinger, Carlo Gambacorti-Passerini, Jianjiong Gao, Shengjie Gao, Levi Garraway, Øystein Garred, Erik Garrison, Dale W. Garsed, Nils Gehlenborg, Josep L. L. Gelpi, Joshy George, Daniela S. Gerhard, Clarissa Gerhauser, Jeffrey E. Gershenwald, Mark Gerstein, Moritz Gerstung, Gad Getz, Mohammed Ghori, Ronald Ghossein, Nasra H. Giama, Richard A. Gibbs, Bob Gibson, Anthony J. Gill, Pelvender Gill, Dilip D. Giri, Dominik Glodzik, Vincent J. Gnanapragasam, Maria Elisabeth Goebler, Mary J. Goldman, Carmen Gomez, Santiago Gonzalez, Abel Gonzalez-Perez, Dmitry A. Gordenin, James Gossage, Kunihito Gotoh, Ramaswamy Govindan, Dorthe Grabau, Janet S. Graham, Robert C. Grant, Anthony R. Green, Eric Green, Liliana Greger, Nicola Grehan, Sonia Grimaldi, Sean M. Grimmond, Robert L. Grossman, Adam Grundhoff, Gunes Gundem, Qianyun Guo, Manaswi Gupta, Shailja Gupta, Ivo G. Gut, Marta Gut, Jonathan Göke, Gavin Ha, Andrea Haake, David Haan, Siegfried Haas, Kerstin Haase, James E. Haber, Nina Habermann, Faraz Hach, Syed Haider, Natsuko Hama, Freddie C. Hamdy, Anne Hamilton, Mark P. Hamilton, Leng Han, George B. Hanna, Martin Hansmann, Nicholas J. Haradhvala, Olivier Harismendy, Ivon Harliwong, Arif O. Harmanci, Eoghan Harrington, Takanori Hasegawa, David Haussler, Steve Hawkins, Shinya Hayami, Shuto Hayashi, D. Neil Hayes, Stephen J. Hayes, Nicholas K. Hayward, Steven Hazell, Yao He, Allison P. Heath, Simon C. Heath, David Hedley, Apurva M. Hegde, David I. Heiman, Michael C. Heinold, Zachary Heins, Lawrence E. Heisler, Eva Hellstrom-Lindberg, Mohamed Helmy, Seong Gu Heo, Austin J. Hepperla, José María Heredia-Genestar, Carl Herrmann, Peter Hersey, Julian M. Hess, Holmfridur Hilmarsdottir, Jonathan Hinton, Satoshi Hirano, Nobuyoshi Hiraoka, Katherine A. Hoadley, Asger Hobolth, Ermin Hodzic, Jessica I. Hoell, Steve Hoffmann, Oliver Hofmann, Andrea Holbrook, Aliaksei Z. Holik, Michael A. Hollingsworth, Oliver Holmes, Robert A. Holt, Chen Hong, Eun Pyo Hong, Jongwhi H. Hong, Gerrit K. Hooijer, Henrik Hornshøj, Fumie Hosoda, Yong Hou, Volker Hovestadt, William Howat, Alan P. Hoyle, Ralph H. Hruban, Jianhong Hu, Taobo Hu, Xing Hua, Kuan-lin Huang, Mei Huang, Mi Ni Huang, Vincent Huang, Yi Huang, Wolfgang Huber, Thomas J. Hudson, Michael Hummel, Jillian A. Hung, David Huntsman, Ted R. Hupp, Jason Huse, Matthew R. Huska, Barbara Hutter, Carolyn M. Hutter, Daniel Hübschmann, Christine A. Iacobuzio-Donahue, Charles David Imbusch, Marcin Imielinski, Seiya Imoto, William B. Isaacs, Keren Isaev, Shumpei Ishikawa, Murat Iskar, S. M. Ashiqul Islam, Michael Ittmann, Sinisa Ivkovic, Jose M. G. Izarzugaza, Jocelyne Jacquemier, Valerie Jakrot, Nigel B. Jamieson, Gun Ho Jang, Se Jin Jang, Joy C. Jayaseelan, Reyka Jayasinghe, Stuart R. Jefferys, Karine Jegalian, Jennifer L. Jennings, Seung-Hyup Jeon, Lara Jerman, Yuan Ji, Wei Jiao, Peter A. Johansson, Amber L. Johns, Jeremy Johns, Rory Johnson, Todd A. Johnson, Clemency Jolly, Yann Joly, Jon G. Jonasson, Corbin D. Jones, David R. Jones, David T. W. Jones, Nic Jones, Steven J. M. Jones, Jos Jonkers, Young Seok Ju, Hartmut Juhl, Jongsun Jung, Malene Juul, Randi Istrup Juul, Sissel Juul, Natalie Jäger, Rolf Kabbe, Andre Kahles, Abdullah Kahraman, Vera B. Kaiser, Hojabr Kakavand, Sangeetha Kalimuthu, Christof von Kalle, Koo Jeong Kang, Katalin Karaszi, Beth Karlan, Rosa Karlić, Dennis Karsch, Katayoon Kasaian, Karin S. Kassahn, Hitoshi Katai, Mamoru Kato, Hiroto Katoh, Yoshiiku Kawakami, Jonathan D. Kay, Stephen H. Kazakoff, Marat D. Kazanov, Maria Keays, Electron Kebebew, Richard F. Kefford, Manolis Kellis, James G. Kench, Catherine J. Kennedy, Jules N. A. Kerssemakers, David Khoo, Vincent Khoo, Narong Khuntikeo, Ekta Khurana, Helena Kilpinen, Hark Kyun Kim, Hyung-Lae Kim, Hyung-Yong Kim, Hyunghwan Kim, Jaegil Kim, Jihoon Kim, Jong K. Kim, Youngwook Kim, Tari A. King, Wolfram Klapper, Kortine Kleinheinz, Leszek J. Klimczak, Stian Knappskog, Michael Kneba, Bartha M. Knoppers, Youngil Koh, Jan Komorowski, Daisuke Komura, Mitsuhiro Komura, Gu Kong, Marcel Kool, Jan O. Korbel, Viktoriya Korchina, Andrey Korshunov, Michael Koscher, Roelof Koster, Zsofia Kote-Jarai, Antonios Koures, Milena Kovacevic, Barbara Kremeyer, Helene Kretzmer, Markus Kreuz, Savitri Krishnamurthy, Dieter Kube, Kiran Kumar, Pardeep Kumar, Sushant Kumar, Yogesh Kumar, Ritika Kundra, Kirsten Kübler, Ralf Küppers, Jesper Lagergren, Phillip H. Lai, Peter W. Laird, Sunil R. Lakhani, Christopher M. Lalansingh, Emilie Lalonde, Fabien C. Lamaze, Adam Lambert, Eric Lander, Pablo Landgraf, Luca Landoni, Anita Langerød, Andrés Lanzós, Denis Larsimont, Erik Larsson, Mark Lathrop, Loretta M. S. Lau, Chris Lawerenz, Rita T. Lawlor, Michael S. Lawrence, Alexander J. Lazar, Ana Mijalkovic Lazic, Xuan Le, Darlene Lee, Donghoon Lee, Eunjung Alice Lee, Hee Jin Lee, Jake June-Koo Lee, Jeong-Yeon Lee, Juhee Lee, Ming Ta Michael Lee, Henry Lee-Six, Kjong-Van Lehmann, Hans Lehrach, Dido Lenze, Conrad R. Leonard, Daniel A. Leongamornlert, Ignaty Leshchiner, Louis Letourneau, Ivica Letunic, Douglas A. Levine, Lora Lewis, Tim Ley, Chang Li, Constance H. Li, Haiyan Irene Li, Jun Li, Lin Li, Shantao Li, Siliang Li, Xiaobo Li, Xiaotong Li, Xinyue Li, Yilong Li, Han Liang, Sheng-Ben Liang, Peter Lichter, Pei Lin, Ziao Lin, W. M. Linehan, Ole Christian Lingjærde, Dongbing Liu, Eric Minwei Liu, Fei-Fei Fei Liu, Fenglin Liu, Jia Liu, Xingmin Liu, Julie Livingstone, Dimitri Livitz, Naomi Livni, Lucas Lochovsky, Markus Loeffler, Georgina V. Long, Armando Lopez-Guillermo, Shaoke Lou, David N. Louis, Laurence B. Lovat, Yiling Lu, Yong-Jie Lu, Youyong Lu, Claudio Luchini, Ilinca Lungu, Xuemei Luo, Hayley J. Luxton, Andy G. Lynch, Lisa Lype, Cristina López, Carlos López-Otín, Eric Z. Ma, Yussanne Ma, Gaetan MacGrogan, Shona MacRae, Geoff Macintyre, Tobias Madsen, Kazuhiro Maejima, Andrea Mafficini, Dennis T. Maglinte, Arindam Maitra, Partha P. Majumder, Luca Malcovati, Salem Malikic, Giuseppe Malleo, Graham J. Mann, Luisa Mantovani-Löffler, Kathleen Marchal, Giovanni Marchegiani, Elaine R. Mardis, Adam A. Margolin, Maximillian G. Marin, Florian Markowetz, Julia Markowski, Jeffrey Marks, Tomas Marques-Bonet, Marco A. Marra, Luke Marsden, John W. M. Martens, Sancha Martin, Jose I. Martin-Subero, Iñigo Martincorena, Alexander Martinez-Fundichely, Yosef E. Maruvka, R. Jay Mashl, Charlie E. Massie, Thomas J. Matthew, Lucy Matthews, Erik Mayer, Simon Mayes, Michael Mayo, Faridah Mbabaali, Karen McCune, Ultan McDermott, Patrick D. McGillivray, Michael D. McLellan, John D. McPherson, John R. McPherson, Treasa A. McPherson, Samuel R. Meier, Alice Meng, Shaowu Meng, Andrew Menzies, Neil D. Merrett, Sue Merson, Matthew Meyerson, William Meyerson, Piotr A. Mieczkowski, George L. Mihaiescu, Sanja Mijalkovic, Tom Mikkelsen, Michele Milella, Linda Mileshkin, Christopher A. Miller, David K. Miller, Jessica K. Miller, Gordon B. Mills, Ana Milovanovic, Sarah Minner, Marco Miotto, Gisela Mir Arnau, Lisa Mirabello, Chris Mitchell, Thomas J. Mitchell, Satoru Miyano, Naoki Miyoshi, Shinichi Mizuno, Fruzsina Molnár-Gábor, Malcolm J. Moore, Richard A. Moore, Sandro Morganella, Quaid D. Morris, Carl Morrison, Lisle E. Mose, Catherine D. Moser, Ferran Muiños, Loris Mularoni, Andrew J. Mungall, Karen Mungall, Elizabeth A. Musgrove, Ville Mustonen, David Mutch, Francesc Muyas, Donna M. Muzny, Alfonso Muñoz, Jerome Myers, Ola Myklebost, Peter Möller, Genta Nagae, Adnan M. Nagrial, Hardeep K. Nahal-Bose, Hitoshi Nakagama, Hidewaki Nakagawa, Hiromi Nakamura, Toru Nakamura, Kaoru Nakano, Tannistha Nandi, Jyoti Nangalia, Mia Nastic, Arcadi Navarro, Fabio C. P. Navarro, David E. Neal, Gerd Nettekoven, Felicity Newell, Steven J. Newhouse, Yulia Newton, Alvin Wei Tian Ng, Anthony Ng, Jonathan Nicholson, David Nicol, Yongzhan Nie, G. Petur Nielsen, Morten Muhlig Nielsen, Serena Nik-Zainal, Michael S. Noble, Katia Nones, Paul A. Northcott, Faiyaz Notta, Brian D. O’Connor, Peter O’Donnell, Maria O’Donovan, Sarah O’Meara, Brian Patrick O’Neill, J. Robert O’Neill, David Ocana, Angelica Ochoa, Layla Oesper, Christopher Ogden, Hideki Ohdan, Kazuhiro Ohi, Lucila Ohno-Machado, Karin A. Oien, Akinyemi I. Ojesina, Hidenori Ojima, Takuji Okusaka, Larsson Omberg, Choon Kiat Ong, Stephan Ossowski, German Ott, B. F. Francis Ouellette, Christine P’ng, Marta Paczkowska, Salvatore Paiella, Chawalit Pairojkul, Marina Pajic, Qiang Pan-Hammarström, Elli Papaemmanuil, Irene Papatheodorou, Nagarajan Paramasivam, Ji Wan Park, Joong-Won Park, Keunchil Park, Kiejung Park, Peter J. Park, Joel S. Parker, Simon L. Parsons, Harvey Pass, Danielle Pasternack, Alessandro Pastore, Ann-Marie Patch, Iris Pauporté, Antonio Pea, John V. Pearson, Chandra Sekhar Pedamallu, Jakob Skou Pedersen, Paolo Pederzoli, Martin Peifer, Nathan A. Pennell, Charles M. Perou, Marc D. Perry, Gloria M. Petersen, Myron Peto, Nicholas Petrelli, Robert Petryszak, Stefan M. Pfister, Mark Phillips, Oriol Pich, Hilda A. Pickett, Todd D. Pihl, Nischalan Pillay, Sarah Pinder, Mark Pinese, Andreia V. Pinho, Esa Pitkänen, Xavier Pivot, Elena Piñeiro-Yáñez, Laura Planko, Christoph Plass, Paz Polak, Tirso Pons, Irinel Popescu, Olga Potapova, Aparna Prasad, Shaun R. Preston, Manuel Prinz, Antonia L. Pritchard, Stephenie D. Prokopec, Elena Provenzano, Xose S. Puente, Sonia Puig, Montserrat Puiggròs, Sergio Pulido-Tamayo, Gulietta M. Pupo, Colin A. Purdie, Michael C. Quinn, Raquel Rabionet, Janet S. Rader, Bernhard Radlwimmer, Petar Radovic, Benjamin Raeder, Keiran M. Raine, Manasa Ramakrishna, Kamna Ramakrishnan, Suresh Ramalingam, Benjamin J. Raphael, W. Kimryn Rathmell, Tobias Rausch, Guido Reifenberger, Jüri Reimand, Jorge Reis-Filho, Victor Reuter, Iker Reyes-Salazar, Matthew A. Reyna, Sheila M. Reynolds, Esther Rheinbay, Yasser Riazalhosseini, Andrea L. Richardson, Julia Richter, Matthew Ringel, Markus Ringnér, Yasushi Rino, Karsten Rippe, Jeffrey Roach, Lewis R. Roberts, Nicola D. Roberts, Steven A. Roberts, A. Gordon Robertson, Alan J. Robertson, Javier Bartolomé Rodriguez, Bernardo Rodriguez-Martin, F. Germán Rodríguez-González, Michael H. A. Roehrl, Marius Rohde, Hirofumi Rokutan, Gilles Romieu, Ilse Rooman, Tom Roques, Daniel Rosebrock, Mara Rosenberg, Philip C. Rosenstiel, Andreas Rosenwald, Edward W. Rowe, Romina Royo, Steven G. Rozen, Yulia Rubanova, Mark A. Rubin, Carlota Rubio-Perez, Vasilisa A. Rudneva, Borislav C. Rusev, Andrea Ruzzenente, Gunnar Rätsch, Radhakrishnan Sabarinathan, Veronica Y. Sabelnykova, Sara Sadeghi, S. Cenk Sahinalp, Natalie Saini, Mihoko Saito-Adachi, Gordon Saksena, Adriana Salcedo, Roberto Salgado, Leonidas Salichos, Richard Sallari, Charles Saller, Roberto Salvia, Michelle Sam, Jaswinder S. Samra, Francisco Sanchez-Vega, Chris Sander, Grant Sanders, Rajiv Sarin, Iman Sarrafi, Aya Sasaki-Oku, Torill Sauer, Guido Sauter, Robyn P. M. Saw, Maria Scardoni, Christopher J. Scarlett, Aldo Scarpa, Ghislaine Scelo, Dirk Schadendorf, Jacqueline E. Schein, Markus B. Schilhabel, Matthias Schlesner, Thorsten Schlomm, Heather K. Schmidt, Sarah-Jane Schramm, Stefan Schreiber, Nikolaus Schultz, Steven E. Schumacher, Roland F. Schwarz, Richard A. Scolyer, David Scott, Ralph Scully, Raja Seethala, Ayellet V. Segre, Iris Selander, Colin A. Semple, Yasin Senbabaoglu, Subhajit Sengupta, Elisabetta Sereni, Stefano Serra, Dennis C. Sgroi, Mark Shackleton, Nimish C. Shah, Sagedeh Shahabi, Catherine A. Shang, Ping Shang, Ofer Shapira, Troy Shelton, Ciyue Shen, Hui Shen, Rebecca Shepherd, Ruian Shi, Yan Shi, Yu-Jia Shiah, Tatsuhiro Shibata, Juliann Shih, Eigo Shimizu, Kiyo Shimizu, Seung Jun Shin, Yuichi Shiraishi, Tal Shmaya, Ilya Shmulevich, Solomon I. Shorser, Charles Short, Raunak Shrestha, Suyash S. Shringarpure, Craig Shriver, Shimin Shuai, Nikos Sidiropoulos, Reiner Siebert, Anieta M. Sieuwerts, Lina Sieverling, Sabina Signoretti, Katarzyna O. Sikora, Michele Simbolo, Ronald Simon, Janae V. Simons, Jared T. Simpson, Peter T. Simpson, Samuel Singer, Nasa Sinnott-Armstrong, Payal Sipahimalani, Tara J. Skelly, Marcel Smid, Jaclyn Smith, Karen Smith-McCune, Nicholas D. Socci, Heidi J. Sofia, Matthew G. Soloway, Lei Song, Anil K. Sood, Sharmila Sothi, Christos Sotiriou, Cameron M. Soulette, Paul N. Span, Paul T. Spellman, Nicola Sperandio, Andrew J. Spillane, Oliver Spiro, Jonathan Spring, Johan Staaf, Peter F. Stadler, Peter Staib, Stefan G. Stark, Lucy Stebbings, Ólafur Andri Stefánsson, Oliver Stegle, Lincoln D. Stein, Alasdair Stenhouse, Chip Stewart, Stephan Stilgenbauer, Miranda D. Stobbe, Michael R. Stratton, Jonathan R. Stretch, Adam J. Struck, Joshua M. Stuart, Henk G. Stunnenberg, Hong Su, Xiaoping Su, Ren X. Sun, Stephanie Sungalee, Hana Susak, Akihiro Suzuki, Fred Sweep, Monika Szczepanowski, Holger Sültmann, Takashi Yugawa, Angela Tam, David Tamborero, Benita Kiat Tee Tan, Donghui Tan, Patrick Tan, Hiroko Tanaka, Hirokazu Taniguchi, Tomas J. Tanskanen, Maxime Tarabichi, Roy Tarnuzzer, Patrick Tarpey, Morgan L. Taschuk, Kenji Tatsuno, Simon Tavaré, Darrin F. Taylor, Amaro Taylor-Weiner, Jon W. Teague, Bin Tean Teh, Varsha Tembe, Javier Temes, Kevin Thai, Sarah P. Thayer, Nina Thiessen, Gilles Thomas, Sarah Thomas, Alan Thompson, Alastair M. Thompson, John F. F. Thompson, R. Houston Thompson, Heather Thorne, Leigh B. Thorne, Adrian Thorogood, Grace Tiao, Nebojsa Tijanic, Lee E. Timms, Roberto Tirabosco, Marta Tojo, Stefania Tommasi, Christopher W. Toon, Umut H. Toprak, David Torrents, Giampaolo Tortora, Jörg Tost, Yasushi Totoki, David Townend, Nadia Traficante, Isabelle Treilleux, Jean-Rémi Trotta, Lorenz H. P. Trümper, Ming Tsao, Tatsuhiko Tsunoda, Jose M. C. Tubio, Olga Tucker, Richard Turkington, Daniel J. Turner, Andrew Tutt, Masaki Ueno, Naoto T. Ueno, Christopher Umbricht, Husen M. Umer, Timothy J. Underwood, Lara Urban, Tomoko Urushidate, Tetsuo Ushiku, Liis Uusküla-Reimand, Alfonso Valencia, David J. Van Den Berg, Steven Van Laere, Peter Van Loo, Erwin G. Van Meir, Gert G. Van den Eynden, Theodorus Van der Kwast, Naveen Vasudev, Miguel Vazquez, Ravikiran Vedururu, Umadevi Veluvolu, Shankar Vembu, Lieven P. C. Verbeke, Peter Vermeulen, Clare Verrill, Alain Viari, David Vicente, Caterina Vicentini, K. VijayRaghavan, Juris Viksna, Ricardo E. Vilain, Izar Villasante, Anne Vincent-Salomon, Tapio Visakorpi, Douglas Voet, Paresh Vyas, Ignacio Vázquez-García, Nick M. Waddell, Nicola Waddell, Claes Wadelius, Lina Wadi, Rabea Wagener, Jeremiah A. Wala, Jian Wang, Jiayin Wang, Linghua Wang, Qi Wang, Wenyi Wang, Yumeng Wang, Zhining Wang, Paul M. Waring, Hans-Jörg Warnatz, Jonathan Warrell, Anne Y. Warren, Sebastian M. Waszak, David C. Wedge, Dieter Weichenhan, Paul Weinberger, John N. Weinstein, Joachim Weischenfeldt, Daniel J. Weisenberger, Ian Welch, Michael C. Wendl, Johannes Werner, Justin P. Whalley, David A. Wheeler, Hayley C. Whitaker, Dennis Wigle, Matthew D. Wilkerson, Ashley Williams, James S. Wilmott, Gavin W. Wilson, Julie M. Wilson, Richard K. Wilson, Boris Winterhoff, Jeffrey A. Wintersinger, Maciej Wiznerowicz, Stephan Wolf, Bernice H. Wong, Tina Wong, Winghing Wong, Youngchoon Woo, Scott Wood, Bradly G. Wouters, Adam J. Wright, Derek W. Wright, Mark H. Wright, Chin-Lee Wu, Dai-Ying Wu, Guanming Wu, Jianmin Wu, Kui Wu, Yang Wu, Zhenggang Wu, Liu Xi, Tian Xia, Qian Xiang, Xiao Xiao, Rui Xing, Heng Xiong, Qinying Xu, Yanxun Xu, Hong Xue, Shinichi Yachida, Sergei Yakneen, Rui Yamaguchi, Takafumi N. Yamaguchi, Masakazu Yamamoto, Shogo Yamamoto, Hiroki Yamaue, Fan Yang, Huanming Yang, Jean Y. Yang, Liming Yang, Lixing Yang, Shanlin Yang, Tsun-Po Yang, Yang Yang, Xiaotong Yao, Marie-Laure Yaspo, Lucy Yates, Christina Yau, Chen Ye, Kai Ye, Venkata D. Yellapantula, Christopher J. Yoon, Sung-Soo Yoon, Fouad Yousif, Jun Yu, Kaixian Yu, Willie Yu, Yingyan Yu, Ke Yuan, Yuan Yuan, Denis Yuen, Christina K. Yung, Olga Zaikova, Jorge Zamora, Marc Zapatka, Jean C. Zenklusen, Thorsten Zenz, Nikolajs Zeps, Cheng-Zhong Zhang, Fan Zhang, Hailei Zhang, Hongwei Zhang, Hongxin Zhang, Jiashan Zhang, Jing Zhang, Junjun Zhang, Xiuqing Zhang, Xuanping Zhang, Yan Zhang, Zemin Zhang, Zhongming Zhao, Liangtao Zheng, Xiuqing Zheng, Wanding Zhou, Yong Zhou, Bin Zhu, Hongtu Zhu, Jingchun Zhu, Shida Zhu, Lihua Zou, Xueqing Zou, Anna deFazio, Nicholas van As, Carolien H. M. van Deurzen, Marc J. van de Vijver, L. van’t Veer, Christian von Mering

**Affiliations:** 1 Department of Genomic Medicine, University of Texas MD Anderson Cancer Center, Houston, TX USA; 2 Salk Institute for Biological Studies, La Jolla, CA USA; 3 Wellcome Trust Sanger Institute, Cambridge, UK; 4 Division of Computational Biology, The Jackson Laboratory for Genomic Medicine, Farmington, CT USA; 5 Department of Medical Oncology, Dana-Farber Cancer Institute, Boston, MA USA; 6 Department of Cancer Biology, Dana-Farber Cancer Institute, Cambridge, MA USA; 7 Broad Institute of MIT and Harvard, Cambridge, MA USA; 8 Harvard Medical School, Boston, MA USA; 9 Department of Haematology, University of Cambridge, Cambridge, UK; 10 Institute for Health Transformation University of Texas, Houston, TX USA; 13 Department of Zoology, Genetics and Physical Anthropology, Universidade de Santiago de Compostela, Santiago de Compostela, Spain; 14 Centre for Research in Molecular Medicine and Chronic Diseases (CIMUS), Universidade de Santiago de Compostela, Santiago de Compostela, Spain; 15 The Biomedical Research Centre (CINBIO), Universidade de Vigo, Vigo, Spain; 16 Transmissible Cancer Group, Department of Veterinary Medicine, University of Cambridge, Cambridge, UK; 17 Computational Biology Program, Ontario Institute for Cancer Research, Toronto, Ontario Canada; 18 Department of Medical Biophysics, University of Toronto, Toronto, Ontario Canada; 19 Department of Pharmacology, University of Toronto, Toronto, Ontario Canada; 20 University of California Los Angeles, Los Angeles, CA USA; 21 Peter MacCallum Cancer Centre, Melbourne, Victoria Australia; 22 Sir Peter MacCallum Department of Oncology, University of Melbourne, Melbourne, Victoria Australia; 23 Division of Applied Bioinformatics, German Cancer Research Center (DKFZ), Heidelberg, Germany; 24 German Cancer Consortium (DKTK), Heidelberg, Germany; 25 National Center for Tumor Diseases (NCT) Heidelberg, Heidelberg, Germany; 26 Johns Hopkins School of Medicine, Baltimore, MD USA; 27 Department of Biochemistry, Microbiology and Immunology, Faculty of Medicine, University of Ottawa, Ottawa, Ontario Canada; 28 University of Texas MD Anderson Cancer Center, Houston, TX USA; 29 Centre for Molecular Science Informatics, Department of Chemistry, University of Cambridge, Cambridge, UK; 30 Department of Biomedical Informatics, Harvard Medical School, Boston, MA USA; 31 Ludwig Center, Harvard Medical School, Boston, MA USA; 32 Barcelona Supercomputing Center (BSC), Barcelona, Spain; 33 Cancer Research UK Cambridge Institute, University of Cambridge, Cambridge, UK; 34 University of Cambridge, Cambridge, UK; 35 Sidra Medicine, Doha, Qatar; 36 Sir Peter MacCallum Department of Oncology, The University of Melbourne, Melbourne, Victoria Australia; 37 Queensland Centre for Medical Genomics, Institute for Molecular Bioscience, The University of Queensland, St. Lucia, Queensland Australia; 38 The Azrieli Faculty of Medicine, Bar-Ilan University, Safed, Israel; 39 Department of Computer Science, Princeton University, Princeton, NJ USA; 40 Department of Computer Science, Yale University, New Haven, CT USA; 41 Department of Molecular Biophysics and Biochemistry, Yale University, New Haven, CT USA; 42 Program in Computational Biology and Bioinformatics, Yale University, New Haven, CT USA; 43 Genome Integrity and Structural Biology Laboratory, National Institute of Environmental Health Sciences (NIEHS), Durham, NC USA; 44 Biomolecular Engineering Department, University of California, Santa Cruz, Santa Cruz, CA USA; 45 Brandeis University, Waltham, MA USA; 46 Massachusetts General Hospital Center for Cancer Research, Charlestown, MA USA; 47 Heidelberg Center for Personalized Oncology (DKFZ-HIPO), German Cancer Research Center (DKFZ), Heidelberg, Germany; 48 New York Genome Center, New York, NY USA; 49 Weill Cornell Medicine, New York, NY USA; 50 Hopp Children’s Cancer Center (KiTZ), Heidelberg, Germany; 51 Pediatric Glioma Research Group, German Cancer Research Center (DKFZ), Heidelberg, Germany; 52 Korea Advanced Institute of Science and Technology, Daejeon, South Korea; 53 Skolkovo Institute of Science and Technology, Moscow, Russia; 54 A. A. Kharkevich Institute of Information Transmission Problems, Moscow, Russia; 55 Dmitry Rogachev National Research Center of Pediatric Hematology, Oncology and Immunology, Moscow, Russia; 56 Integrative Bioinformatics Support Group, National Institute of Environmental Health Sciences (NIEHS), Durham, NC USA; 57 Center For Medical Innovation, Seoul National University Hospital, Seoul, South Korea; 58 Department of Internal Medicine, Seoul National University Hospital, Seoul, South Korea; 59 European Molecular Biology Laboratory, European Bioinformatics Institute (EMBL-EBI), Cambridge, UK; 60 Genome Biology Unit, European Molecular Biology Laboratory (EMBL), Heidelberg, Germany; 61 Division of Genetics and Genomics, Boston Children’s Hospital and Harvard Medical School, Boston, MA USA; 62 Ludwig Center at Harvard, Boston, MA USA; 63 School of Medicine/School of Mathematics and Statistics, University of St. Andrews, St. Andrews, UK; 64 Department of Physiology and Biophysics, Weill Cornell Medicine, New York, NY USA; 65 Englander Institute for Precision Medicine, Weill Cornell Medicine, New York, NY USA; 66 Institute for Computational Biomedicine, Weill Cornell Medicine, New York, NY USA; 67 Dana-Farber Cancer Institute, Boston, MA USA; 68 Department of Medical Oncology, Inselspital, University Hospital and University of Bern, Bern, Switzerland; 69 Department of Pathology, The University of Melbourne, Melbourne, Victoria Australia; 70 The Institute of Medical Science, The University of Tokyo, Tokyo, Japan; 71 RIKEN Center for Integrative Medical Sciences, Yokohama, Japan; 72 Centre for Genomic Regulation (CRG), The Barcelona Institute of Science and Technology, Barcelona, Spain; 73 Institute of Medical Genetics and Applied Genomics, University of Tübingen, Tübingen, Germany; 74 Universitat Pompeu Fabra (UPF), Barcelona, Spain; 75 Department of Genetics and Computational Biology, QIMR Berghofer Medical Research Institute, Brisbane, Queensland Australia; 76 Institute for Molecular Bioscience, University of Queensland, St. Lucia, Queensland Australia; 77 German Cancer Research Center (DKFZ), Heidelberg, Germany; 78 School of Molecular Biosciences and Center for Reproductive Biology, Washington State University, Pullman, WA USA; 79 Department of Cancer Biology, Dana-Farber Cancer Institute, Boston, MA USA; 80 Cancer Research Institute, Beth Israel Deaconess Medical Center, Boston, MA USA; 81 Peter MacCallum Cancer Centre and University of Melbourne, Melbourne, Victoria Australia; 82 Finsen Laboratory and Biotech Research & Innovation Centre (BRIC), University of Copenhagen, Copenhagen, Denmark; 83 Faculty of Biosciences, Heidelberg University, Heidelberg, Germany; 84 Institució Catalana de Recerca i Estudis Avançats (ICREA), Barcelona, Spain; 85 Department of Urology, Charité Universitätsmedizin Berlin, Berlin, Germany; 86 Ben May Department for Cancer Research, Department of Human Genetics, The University of Chicago, Chicago, IL USA; 87 Tri-institutional PhD program of Computational Biology and Medicine, Weill Cornell Medicine, New York, NY USA; 200 Applied Tumor Genomics Research Program, Research Programs Unit, University of Helsinki, Helsinki, Finland; 201 Wellcome Sanger Institute, Wellcome Genome Campus, Hinxton, UK; 202 Memorial Sloan Kettering Cancer Center, New York, NY USA; 203 Genome Science Division, Research Center for Advanced Science and Technology, University of Tokyo, Tokyo, Japan; 204 Department of Surgery, University of Chicago, Chicago, IL USA; 205 Department of Surgery, Division of Hepatobiliary and Pancreatic Surgery, School of Medicine, Keimyung University Dongsan Medical Center, Daegu, South Korea; 206 Department of Oncology, Gil Medical Center, Gachon University, Incheon, South Korea; 207 Hiroshima University, Hiroshima, Japan; 208 Department of Bioinformatics and Computational Biology, The University of Texas MD Anderson Cancer Center, Houston, TX USA; 209 University of Texas MD Anderson Cancer Center, Houston, TX USA; 210 King Faisal Specialist Hospital and Research Centre, Al Maather, Riyadh, Saudi Arabia; 211 Bioinformatics Unit, Spanish National Cancer Research Centre (CNIO), Madrid, Spain; 212 Bioinformatics Core Facility, University Medical Center Hamburg, Hamburg, Germany; 213 Heinrich Pette Institute, Leibniz Institute for Experimental Virology, Hamburg, Germany; 214 Ontario Tumour Bank, Ontario Institute for Cancer Research, Toronto, ON Canada; 215 Department of Pathology, The University of Texas MD Anderson Cancer Center, Houston, TX USA; 216 Laboratory of Pathology, Center for Cancer Research, National Cancer Institute, Bethesda, MD USA; 217 Department of Cellular and Molecular Medicine and Department of Bioengineering, University of California San Diego, La Jolla, CA USA; 218 UC San Diego Moores Cancer Center, San Diego, CA USA; 219 Canada’s Michael Smith Genome Sciences Centre, BC Cancer, Vancouver, BC Canada; 220 Sir Peter MacCallum Department of Oncology, Peter MacCallum Cancer Centre, University of Melbourne, Melbourne, VIC Australia; 221 Centre for Research in Molecular Medicine and Chronic Diseases (CiMUS), Universidade de Santiago de Compostela, Santiago de Compostela, Spain; 222 Department of Zoology, Genetics and Physical Anthropology, (CiMUS), Universidade de Santiago de Compostela, Santiago de Compostela, Spain; 223 The Biomedical Research Centre (CINBIO), Universidade de Vigo, Vigo, Spain; 224 Royal National Orthopaedic Hospital - Bolsover, London, UK; 225 Department of Genomic Medicine, The University of Texas MD Anderson Cancer Center, Houston, TX USA; 226 Quantitative and Computational Biosciences Graduate Program, Baylor College of Medicine, Houston, TX USA; 227 The Jackson Laboratory for Genomic Medicine, Farmington, CT USA; 228 Genome Informatics Program, Ontario Institute for Cancer Research, Toronto, ON Canada; 229 Institute of Human Genetics, Christian-Albrechts-University, Kiel, Germany; 230 Institute of Human Genetics, Ulm University and Ulm University Medical Center, Ulm, Germany; 231 Queensland Centre for Medical Genomics, Institute for Molecular Bioscience, University of Queensland, St. Lucia, Brisbane, QLD Australia; 232 Salford Royal NHS Foundation Trust, Salford, UK; 233 Department of Surgery, Pancreas Institute, University and Hospital Trust of Verona, Verona, Italy; 234 Molecular and Medical Genetics, OHSU Knight Cancer Institute, Oregon Health and Science University, Portland, OR USA; 235 Department of Molecular Oncology, BC Cancer Research Centre, Vancouver, BC Canada; 236 The McDonnell Genome Institute at Washington University, St. Louis, MO USA; 237 University College London, London, UK; 238 Division of Cancer Genomics, National Cancer Center Research Institute, National Cancer Center, Tokyo, Japan; 239 DLR Project Management Agency, Bonn, Germany; 240 Tokyo Women’s Medical University, Tokyo, Japan; 241 Center for Molecular Oncology, Memorial Sloan Kettering Cancer Center, New York, NY USA; 242 Los Alamos National Laboratory, Los Alamos, NM USA; 243 Department of Pathology, University Health Network, Toronto General Hospital, Toronto, ON Canada; 244 Nottingham University Hospitals NHS Trust, Nottingham, UK; 245 Epigenomics and Cancer Risk Factors, German Cancer Research Center (DKFZ), Heidelberg, Germany; 246 Computational Biology Program, Ontario Institute for Cancer Research, Toronto, ON Canada; 247 Department of Molecular Genetics, University of Toronto, Toronto, ON Canada; 248 Vector Institute, Toronto, ON Canada; 249 Hematopathology Section, Institute of Pathology, Christian-Albrechts-University, Kiel, Germany; 250 Department of Pathology and Laboratory Medicine, School of Medicine, University of North Carolina at Chapel Hill, Chapel Hill, NC USA; 251 Department of Cancer Genetics, Institute for Cancer Research, Oslo University Hospital, The Norwegian Radium Hospital, Oslo, Norway; 252 Pathology, Hospital Clinic, Institut d’Investigacions Biomèdiques August Pi i Sunyer (IDIBAPS), University of Barcelona, Barcelona, Spain; 253 Department of Veterinary Medicine, Transmissible Cancer Group, University of Cambridge, Cambridge, UK; 254 Alvin J. Siteman Cancer Center, Washington University School of Medicine, St. Louis, MO USA; 255 Wolfson Wohl Cancer Research Centre, Institute of Cancer Sciences, University of Glasgow, Glasgow, UK; 256 Lineberger Comprehensive Cancer Center, University of North Carolina at Chapel Hill, Chapel Hill, NC USA; 257 Broad Institute of MIT and Harvard, Cambridge, MA USA; 258 Dana-Farber/Boston Children’s Cancer and Blood Disorders Center, Boston, MA USA; 259 Department of Pediatrics, Harvard Medical School, Boston, MA USA; 260 Leeds Institute of Medical Research @ St. James’s, University of Leeds, St. James’s University Hospital, Leeds, UK; 261 Department of Pathology and Diagnostics, University and Hospital Trust of Verona, Verona, Italy; 262 Department of Surgery, Princess Alexandra Hospital, Brisbane, QLD Australia; 263 Surgical Oncology Group, Diamantina Institute, University of Queensland, Brisbane, QLD Australia; 264 Department of Population and Quantitative Health Sciences, Case Western Reserve University School of Medicine, Cleveland, OH USA; 265 Research Health Analytics and Informatics, University Hospitals Cleveland Medical Center, Cleveland, OH USA; 266 Gloucester Royal Hospital, Gloucester, UK; 267 European Molecular Biology Laboratory, European Bioinformatics Institute (EMBL-EBI), Cambridge, UK; 268 Diagnostic Development, Ontario Institute for Cancer Research, Toronto, ON Canada; 269 Barcelona Supercomputing Center (BSC), Barcelona, Spain; 270 Arnie Charbonneau Cancer Institute, University of Calgary, Calgary, AB Canada; 271 Departments of Surgery and Oncology, University of Calgary, Calgary, AB Canada; 272 Department of Pathology, Oslo University Hospital, The Norwegian Radium Hospital, Oslo, Norway; 273 PanCuRx Translational Research Initiative, Ontario Institute for Cancer Research, Toronto, ON Canada; 274 Department of Oncology, Sidney Kimmel Comprehensive Cancer Center at Johns Hopkins University School of Medicine, Baltimore, MD USA; 275 University Hospital Southampton NHS Foundation Trust, Southampton, UK; 276 Royal Stoke University Hospital, Stoke-on-Trent, UK; 277 Genome Sequence Informatics, Ontario Institute for Cancer Research, Toronto, ON Canada; 278 Human Longevity Inc, San Diego, CA USA; 279 Olivia Newton-John Cancer Research Institute, La Trobe University, Heidelberg, VIC Australia; 280 Computer Network Information Center, Chinese Academy of Sciences, Beijing, China; 281 Genome Canada, Ottawa, ON Canada; 282 CNAG-CRG, Centre for Genomic Regulation (CRG), Barcelona Institute of Science and Technology (BIST), Barcelona, Spain; 283 Universitat Pompeu Fabra (UPF), Barcelona, Spain; 284 Buck Institute for Research on Aging, Novato, CA USA; 285 Duke University Medical Center, Durham, NC USA; 286 Department of Human Genetics, Hannover Medical School, Hannover, Germany; 287 Center for Bioinformatics and Functional Genomics, Cedars-Sinai Medical Center, Los Angeles, CA USA; 288 Department of Biomedical Sciences, Cedars-Sinai Medical Center, Los Angeles, CA USA; 289 The Hebrew University Faculty of Medicine, Jerusalem, Israel; 290 Barts Cancer Institute, Barts and the London School of Medicine and Dentistry, Queen Mary University of London, London, UK; 291 Department of Computer Science, Bioinformatics Group, University of Leipzig, Leipzig, Germany; 292 Interdisciplinary Center for Bioinformatics, University of Leipzig, Leipzig, Germany; 293 Transcriptome Bioinformatics, LIFE Research Center for Civilization Diseases, University of Leipzig, Leipzig, Germany; 294 Department of Medical Oncology, Dana-Farber Cancer Institute, Boston, MA USA; 295 Department of Cancer Biology, Dana-Farber Cancer Institute, Boston, MA USA; 296 Harvard Medical School, Boston, MA USA; 297 USC Norris Comprehensive Cancer Center, University of Southern California, Los Angeles, CA USA; 298 Department of Diagnostics and Public Health, University and Hospital Trust of Verona, Verona, Italy; 299 Department of Mathematics, Aarhus University, Aarhus, Denmark; 300 Department of Molecular Medicine (MOMA), Aarhus University Hospital, Aarhus N, Denmark; 301 Instituto Carlos Slim de la Salud, Mexico City, Mexico; 302 Department of Medical Biophysics, University of Toronto, Toronto, ON Canada; 303 Cancer Division, Garvan Institute of Medical Research, Kinghorn Cancer Centre, University of New South Wales (UNSW Sydney), Sydney, NSW Australia; 304 South Western Sydney Clinical School, Faculty of Medicine, University of New South Wales (UNSW Sydney), Liverpool, NSW Australia; 305 West of Scotland Pancreatic Unit, Glasgow Royal Infirmary, Glasgow, UK; 306 Center for Digital Health, Berlin Institute of Health and Charitè - Universitätsmedizin Berlin, Berlin, Germany; 307 Heidelberg Center for Personalized Oncology (DKFZ-HIPO), German Cancer Research Center (DKFZ), Heidelberg, Germany; 308 The Preston Robert Tisch Brain Tumor Center, Duke University Medical Center, Durham, NC USA; 309 Massachusetts General Hospital, Boston, MA USA; 310 National Institute of Biomedical Genomics, Kalyani, West Bengal India; 311 Institute of Clinical Medicine and Institute of Oral Biology, University of Oslo, Oslo, Norway; 312 University of North Carolina at Chapel Hill, Chapel Hill, NC USA; 313 ARC-Net Centre for Applied Research on Cancer, University and Hospital Trust of Verona, Verona, Italy; 314 The Institute of Cancer Research, London, UK; 315 Centre for Computational Biology, Duke-NUS Medical School, Singapore, Singapore; 316 Programme in Cancer and Stem Cell Biology, Duke-NUS Medical School, Singapore, Singapore; 317 Division of Oncology and Pathology, Department of Clinical Sciences Lund, Lund University, Lund, Sweden; 318 Department of Pediatric Oncology, Hematology and Clinical Immunology, Heinrich-Heine-University, Düsseldorf, Germany; 319 Laboratory for Medical Science Mathematics, RIKEN Center for Integrative Medical Sciences, Yokohama, Japan; 320 RIKEN Center for Integrative Medical Sciences, Yokohama, Japan; 321 Department of Internal Medicine/Hematology, Friedrich-Ebert-Hospital, Neumünster, Germany; 322 Departments of Dermatology and Pathology, Yale University, New Haven, CT USA; 323 Centre for Genomic Regulation (CRG), The Barcelona Institute of Science and Technology, Barcelona, Spain; 324 Radcliffe Department of Medicine, University of Oxford, Oxford, UK; 325 Canadian Center for Computational Genomics, McGill University, Montreal, QC Canada; 326 Department of Human Genetics, McGill University, Montreal, QC Canada; 327 Department of Human Genetics, University of California Los Angeles, Los Angeles, CA USA; 328 Department of Pharmacology, University of Toronto, Toronto, ON Canada; 329 Faculty of Medicine and Health Technology, Tampere University and Tays Cancer Center, Tampere University Hospital, Tampere, Finland; 330 Haematology, Leeds Teaching Hospitals NHS Trust, Leeds, UK; 331 Translational Research and Innovation, Centre Léon Bérard, Lyon, France; 332 Fox Chase Cancer Center, Philadelphia, PA USA; 333 International Agency for Research on Cancer, World Health Organization, Lyon, France; 334 Earlham Institute, Norwich, UK; 335 Norwich Medical School, University of East Anglia, Norwich, UK; 336 Department of Molecular Biology, Faculty of Science, Radboud Institute for Molecular Life Sciences, Radboud University, Nijmegen, HB The Netherlands; 337 CRUK Manchester Institute and Centre, Manchester, UK; 338 Department of Radiation Oncology, University of Toronto, Toronto, ON Canada; 339 Division of Cancer Sciences, Manchester Cancer Research Centre, University of Manchester, Manchester, UK; 340 Radiation Medicine Program, Princess Margaret Cancer Centre, Toronto, ON Canada; 341 Department of Pathology, Brigham and Women’s Hospital, Harvard Medical School, Boston, MA USA; 342 Department of Surgery, Division of Thoracic Surgery, The Johns Hopkins University School of Medicine, Baltimore, MD USA; 343 Division of Molecular Pathology, The Netherlands Cancer Institute, Oncode Institute, Amsterdam, CX The Netherlands; 344 Department of Biomolecular Engineering, University of California Santa Cruz, Santa Cruz, CA USA; 345 UC Santa Cruz Genomics Institute, University of California Santa Cruz, Santa Cruz, CA USA; 346 Division of Applied Bioinformatics, German Cancer Research Center (DKFZ), Heidelberg, Germany; 347 German Cancer Consortium (DKTK), German Cancer Research Center (DKFZ), Heidelberg, Germany; 348 National Center for Tumor Diseases (NCT) Heidelberg, Heidelberg, Germany; 349 Center for Biological Sequence Analysis, Department of Bio and Health Informatics, Technical University of Denmark, Lyngby, Denmark; 350 Novo Nordisk Foundation Center for Protein Research, University of Copenhagen, Copenhagen, Denmark; 351 Institute for Molecular Bioscience, University of Queensland, St. Lucia, Brisbane, QLD Australia; 352 Biomedical Engineering, Oregon Health and Science University, Portland, OR USA; 353 Division of Theoretical Bioinformatics, German Cancer Research Center (DKFZ), Heidelberg, Germany; 354 Institute of Pharmacy and Molecular Biotechnology and BioQuant, Heidelberg University, Heidelberg, Germany; 355 Federal Ministry of Education and Research, Berlin, Germany; 356 Melanoma Institute Australia, University of Sydney, Sydney, NSW Australia; 357 Pediatric Hematology and Oncology, University Hospital Muenster, Muenster, Germany; 358 Department of Pathology, Johns Hopkins University School of Medicine, Baltimore, MD USA; 359 McKusick-Nathans Institute of Genetic Medicine, Sidney Kimmel Comprehensive Cancer Center at Johns Hopkins University School of Medicine, Baltimore, MD USA; 360 Foundation Medicine, Inc, Cambridge, MA USA; 361 Department of Biomedical Data Science, Stanford University School of Medicine, Stanford, CA USA; 362 Department of Genetics, Stanford University School of Medicine, Stanford, CA USA; 363 Bakar Computational Health Sciences Institute and Department of Pediatrics, University of California, San Francisco, CA USA; 364 Institute of Clinical Medicine, Faculty of Medicine, University of Oslo, Oslo, Norway; 365 National Cancer Institute, National Institutes of Health, Bethesda, MD USA; 366 Royal Marsden NHS Foundation Trust, London and Sutton, UK; 367 Genome Biology Unit, European Molecular Biology Laboratory (EMBL), Heidelberg, Germany; 368 Department of Oncology, University of Cambridge, Cambridge, UK; 369 Li Ka Shing Centre, Cancer Research UK Cambridge Institute, University of Cambridge, Cambridge, UK; 370 Institut Gustave Roussy, Villejuif, France; 371 Cambridge University Hospitals NHS Foundation Trust, Cambridge, UK; 372 Department of Haematology, University of Cambridge, Cambridge, UK; 373 Anatomia Patológica, Hospital Clinic, Institut d’Investigacions Biomèdiques August Pi i Sunyer (IDIBAPS), University of Barcelona, Barcelona, Spain; 374 Spanish Ministry of Science and Innovation, Madrid, Spain; 375 University of Michigan Comprehensive Cancer Center, Ann Arbor, MI USA; 376 Department for BioMedical Research, University of Bern, Bern, Switzerland; 377 Department of Medical Oncology, Inselspital, University Hospital and University of Bern, Bern, Switzerland; 378 Graduate School for Cellular and Biomedical Sciences, University of Bern, Bern, Switzerland; 379 University of Pavia, Pavia, Italy; 380 University of Alabama at Birmingham, Birmingham, AL USA; 381 UHN Program in BioSpecimen Sciences, Toronto General Hospital, Toronto, ON Canada; 382 Department of Urology, Icahn School of Medicine at Mount Sinai, New York, NY USA; 383 Centre for Law and Genetics, University of Tasmania, Sandy Bay Campus, Hobart, TAS Australia; 384 Faculty of Biosciences, Heidelberg University, Heidelberg, Germany; 385 Department of Biochemistry, Microbiology and Immunology, Faculty of Medicine, University of Ottawa, Ottawa, ON Canada; 386 Division of Anatomic Pathology, Mayo Clinic, Rochester, MN USA; 387 Division of Cancer Epidemiology and Genetics, National Cancer Institute, National Institutes of Health, Bethesda, MD USA; 388 Illawarra Shoalhaven Local Health District L3 Illawarra Cancer Care Centre, Wollongong Hospital, Wollongong, NSW Australia; 389 BioForA, French National Institute for Agriculture, Food, and Environment (INRAE), ONF, Orléans, France; 390 Department of Biostatistics, Bloomberg School of Public Health, Johns Hopkins University, Baltimore, MD USA; 391 University of California San Diego, San Diego, CA USA; 392 Division of Experimental Pathology, Mayo Clinic, Rochester, MN USA; 393 Centre for Cancer Research, The Westmead Institute for Medical Research, University of Sydney, Sydney, NSW Australia; 394 Department of Gynaecological Oncology, Westmead Hospital, Sydney, NSW Australia; 395 PDXen Biosystems Inc, Seoul, South Korea; 396 Korea Advanced Institute of Science and Technology, Daejeon, South Korea; 397 Electronics and Telecommunications Research Institute, Daejeon, South Korea; 398 Institut National du Cancer (INCA), Boulogne-Billancourt, France; 399 Department of Genetics, Informatics Institute, University of Alabama at Birmingham, Birmingham, AL USA; 400 Division of Medical Oncology, National Cancer Centre, Singapore, Singapore; 401 Medical Oncology, University and Hospital Trust of Verona, Verona, Italy; 402 Department of Pediatrics, University Hospital Schleswig-Holstein, Kiel, Germany; 403 Hepatobiliary/Pancreatic Surgical Oncology Program, University Health Network, Toronto, ON Canada; 404 School of Biological Sciences, University of Auckland, Auckland, New Zealand; 405 Department of Surgery, University of Melbourne, Parkville, VIC Australia; 406 The Murdoch Children’s Research Institute, Royal Children’s Hospital, Parkville, VIC Australia; 407 Walter and Eliza Hall Institute, Parkville, VIC Australia; 408 Vancouver Prostate Centre, Vancouver, Canada; 409 Lunenfeld-Tanenbaum Research Institute, Mount Sinai Hospital, Toronto, ON Canada; 410 University of East Anglia, Norwich, UK; 411 Norfolk and Norwich University Hospital NHS Trust, Norwich, UK; 412 Victorian Institute of Forensic Medicine, Southbank, VIC Australia; 413 Department of Biomedical Informatics, Harvard Medical School, Boston, MA USA; 414 Department of Chemistry, Centre for Molecular Science Informatics, University of Cambridge, Cambridge, UK; 415 Ludwig Center at Harvard Medical School, Boston, MA USA; 416 Human Genome Sequencing Center, Baylor College of Medicine, Houston, TX USA; 417 Peter MacCallum Cancer Centre, University of Melbourne, Melbourne, VIC Australia; 418 Physics Division, Optimization and Systems Biology Lab, Massachusetts General Hospital, Boston, MA USA; 419 Department of Medicine, Baylor College of Medicine, Houston, TX USA; 420 University of Cologne, Cologne, Germany; 421 International Genomics Consortium, Phoenix, AZ USA; 422 Genomics Research Program, Ontario Institute for Cancer Research, Toronto, ON Canada; 423 Barking Havering and Redbridge University Hospitals NHS Trust, Romford, UK; 424 Children’s Hospital at Westmead, University of Sydney, Sydney, NSW Australia; 425 Department of Medicine, Section of Endocrinology, University and Hospital Trust of Verona, Verona, Italy; 426 Computational Biology Center, Memorial Sloan Kettering Cancer Center, New York, NY USA; 427 Department of Biology, ETH Zurich, Zürich, Switzerland; 428 Department of Computer Science, ETH Zurich, Zurich, Switzerland; 429 SIB Swiss Institute of Bioinformatics, Lausanne, Switzerland; 430 Weill Cornell Medical College, New York, NY USA; 431 Academic Department of Medical Genetics, University of Cambridge, Addenbrooke’s Hospital, Cambridge, UK; 432 MRC Cancer Unit, University of Cambridge, Cambridge, UK; 433 Departments of Pediatrics and Genetics, University of North Carolina at Chapel Hill, Chapel Hill, NC USA; 434 Seven Bridges Genomics, Charlestown, MA USA; 435 Annai Systems, Inc, Carlsbad, CA USA; 436 Department of Pathology, General Hospital of Treviso, Department of Medicine, University of Padua, Treviso, Italy; 437 Department of Computational Biology, University of Lausanne, Lausanne, Switzerland; 438 Department of Genetic Medicine and Development, University of Geneva Medical School, Geneva, CH Switzerland; 439 Swiss Institute of Bioinformatics, University of Geneva, Geneva, CH Switzerland; 440 The Francis Crick Institute, London, UK; 441 University of Leuven, Leuven, Belgium; 442 Institute of Medical Genetics and Applied Genomics, University of Tübingen, Tübingen, Germany; 443 Computational and Systems Biology, Genome Institute of Singapore, Singapore, Singapore; 444 School of Computing, National University of Singapore, Singapore, Singapore; 445 Big Data Institute, Li Ka Shing Centre, University of Oxford, Oxford, UK; 446 Biomedical Data Science Laboratory, Francis Crick Institute, London, UK; 447 Bioinformatics Group, Department of Computer Science, University College London, London, UK; 448 The Edward S. Rogers Sr. Department of Electrical and Computer Engineering, University of Toronto, Toronto, ON Canada; 449 Breast Cancer Translational Research Laboratory JC Heuson, Institut Jules Bordet, Brussels, Belgium; 450 Department of Oncology, Laboratory for Translational Breast Cancer Research, KU Leuven, Leuven, Belgium; 451 Institute for Research in Biomedicine (IRB Barcelona), The Barcelona Institute of Science and Technology, Barcelona, Spain; 452 Research Program on Biomedical Informatics, Universitat Pompeu Fabra, Barcelona, Spain; 453 Division of Medical Oncology, Princess Margaret Cancer Centre, Toronto, ON Canada; 454 Department of Physiology and Biophysics, Weill Cornell Medicine, New York, NY USA; 455 Institute for Computational Biomedicine, Weill Cornell Medicine, New York, NY USA; 456 Department of Pathology, UPMC Shadyside, Pittsburgh, PA USA; 457 Independent Consultant, Wellesley, USA; 458 Department of Cell and Molecular Biology, Science for Life Laboratory, Uppsala University, Uppsala, Sweden; 459 Department of Medicine and Department of Genetics, Washington University School of Medicine, St. Louis, St. Louis, MO USA; 460 Hefei University of Technology, Anhui, China; 461 Translational Cancer Research Unit, GZA Hospitals St.-Augustinus, Center for Oncological Research, Faculty of Medicine and Health Sciences, University of Antwerp, Antwerp, Belgium; 462 Simon Fraser University, Burnaby, BC Canada; 463 University of Pennsylvania, Philadelphia, PA USA; 464 Faculty of Science and Technology, University of Vic—Central University of Catalonia (UVic-UCC), Vic, Spain; 465 The Wellcome Trust, London, UK; 466 The Hospital for Sick Children, Toronto, ON Canada; 467 Department of Pathology, Queen Elizabeth University Hospital, Glasgow, UK; 468 Department of Genetics and Computational Biology, QIMR Berghofer Medical Research Institute, Brisbane, QLD Australia; 469 Department of Oncology, Centre for Cancer Genetic Epidemiology, University of Cambridge, Cambridge, UK; 470 Department of Public Health and Primary Care, Centre for Cancer Genetic Epidemiology, University of Cambridge, Cambridge, UK; 471 Prostate Cancer Canada, Toronto, ON Canada; 472 University of Cambridge, Cambridge, UK; 473 Department of Laboratory Medicine, Translational Cancer Research, Lund University Cancer Center at Medicon Village, Lund University, Lund, Sweden; 474 Heidelberg University, Heidelberg, Germany; 475 New BIH Digital Health Center, Berlin Institute of Health (BIH) and Charité - Universitätsmedizin Berlin, Berlin, Germany; 476 CIBER Epidemiología y Salud Pública (CIBERESP), Madrid, Spain; 477 Research Group on Statistics, Econometrics and Health (GRECS), UdG, Barcelona, Spain; 478 Quantitative Genomics Laboratories (qGenomics), Barcelona, Spain; 479 Icelandic Cancer Registry, Icelandic Cancer Society, Reykjavik, Iceland; 480 State Key Laboratory of Cancer Biology, and Xijing Hospital of Digestive Diseases, Fourth Military Medical University, Shaanxi, China; 481 Department of Medicine (DIMED), Surgical Pathology Unit, University of Padua, Padua, Italy; 482 Rigshospitalet, Copenhagen, Denmark; 483 Center for Cancer Genomics, National Cancer Institute, National Institutes of Health, Bethesda, MD USA; 484 Department of Biochemistry and Molecular Medicine, University of Montreal, Montreal, QC Canada; 485 Australian Institute of Tropical Health and Medicine, James Cook University, Douglas, QLD Australia; 486 Department of Neuro-Oncology, Istituto Neurologico Besta, Milano, Italy; 487 Bioplatforms Australia, North Ryde, NSW Australia; 488 Department of Pathology (Research), University College London Cancer Institute, London, UK; 489 Department of Surgical Oncology, Princess Margaret Cancer Centre, Toronto, ON Canada; 490 Department of Medical Oncology, Josephine Nefkens Institute and Cancer Genomics Centre, Erasmus Medical Center, Rotterdam, CN The Netherlands; 491 The University of Queensland Thoracic Research Centre, The Prince Charles Hospital, Brisbane, QLD Australia; 492 CIBIO/InBIO - Research Center in Biodiversity and Genetic Resources, Universidade do Porto, Vairão, Portugal; 493 HCA Laboratories, London, UK; 494 University of Liverpool, Liverpool, UK; 495 The Azrieli Faculty of Medicine, Bar-Ilan University, Safed, Israel; 496 Department of Neurosurgery, University of Florida, Gainesville, FL USA; 497 Department of Pathology, Graduate School of Medicine, University of Tokyo, Tokyo, Japan; 498 University of Milano Bicocca, Monza, Italy; 499 BGI-Shenzhen, Shenzhen, China; 500 Department of Pathology, Oslo University Hospital Ulleval, Oslo, Norway; 501 Center for Biomedical Informatics, Harvard Medical School, Boston, MA USA; 502 Department Biochemistry and Molecular Biomedicine, University of Barcelona, Barcelona, Spain; 503 Office of Cancer Genomics, National Cancer Institute, National Institutes of Health, Bethesda, MD USA; 504 Cancer Epigenomics, German Cancer Research Center (DKFZ), Heidelberg, Germany; 505 Department of Cancer Biology, The University of Texas MD Anderson Cancer Center, Houston, TX USA; 506 Department of Surgical Oncology, The University of Texas MD Anderson Cancer Center, Houston, TX USA; 507 Department of Computer Science, Yale University, New Haven, CT USA; 508 Department of Molecular Biophysics and Biochemistry, Yale University, New Haven, CT USA; 509 Program in Computational Biology and Bioinformatics, Yale University, New Haven, CT USA; 510 Center for Cancer Research, Massachusetts General Hospital, Boston, MA USA; 511 Department of Pathology, Massachusetts General Hospital, Boston, MA USA; 512 Department of Pathology, Memorial Sloan Kettering Cancer Center, New York, NY USA; 513 Division of Gastroenterology and Hepatology, Mayo Clinic, Rochester, MN USA; 514 University of Sydney, Sydney, NSW Australia; 515 University of Oxford, Oxford, UK; 516 Department of Surgery, Academic Urology Group, University of Cambridge, Cambridge, UK; 517 Department of Medicine II, University of Würzburg, Wuerzburg, Germany; 518 Sylvester Comprehensive Cancer Center, University of Miami, Miami, FL USA; 519 Institut Hospital del Mar d’Investigacions Mèdiques (IMIM), Barcelona, Spain; 520 Genome Integrity and Structural Biology Laboratory, National Institute of Environmental Health Sciences (NIEHS), Durham, NC USA; 521 St. Thomas’s Hospital, London, UK; 522 Osaka International Cancer Center, Osaka, Japan; 523 Department of Pathology, Skåne University Hospital, Lund University, Lund, Sweden; 524 Department of Medical Oncology, Beatson West of Scotland Cancer Centre, Glasgow, UK; 525 National Human Genome Research Institute, National Institutes of Health, Bethesda, MD USA; 526 Centre for Cancer Research, Victorian Comprehensive Cancer Centre, University of Melbourne, Melbourne, VIC Australia; 527 Department of Medicine, Section of Hematology/Oncology, University of Chicago, Chicago, IL USA; 528 German Center for Infection Research (DZIF), Partner Site Hamburg-Borstel-Lübeck-Riems, Hamburg, Germany; 529 Bioinformatics Research Centre (BiRC), Aarhus University, Aarhus, Denmark; 530 Department of Biotechnology, Ministry of Science and Technology, Government of India, New Delhi, Delhi India; 531 National Cancer Centre Singapore, Singapore, Singapore; 532 Brandeis University, Waltham, MA USA; 533 Department of Urologic Sciences, University of British Columbia, Vancouver, BC Canada; 534 Department of Internal Medicine, Stanford University, Stanford, CA USA; 535 The University of Texas Health Science Center at Houston, Houston, TX USA; 536 Imperial College NHS Trust, Imperial College, London, INY UK; 537 Senckenberg Institute of Pathology, University of Frankfurt Medical School, Frankfurt, Germany; 538 Department of Medicine, Division of Biomedical Informatics, UC San Diego School of Medicine, San Diego, CA USA; 539 Center for Precision Health, School of Biomedical Informatics, The University of Texas Health Science Center, Houston, TX USA; 540 Oxford Nanopore Technologies, New York, NY USA; 541 Institute of Medical Science, University of Tokyo, Tokyo, Japan; 542 Howard Hughes Medical Institute, University of California Santa Cruz, Santa Cruz, CA USA; 543 Wakayama Medical University, Wakayama, Japan; 544 Department of Internal Medicine, Division of Medical Oncology, Lineberger Comprehensive Cancer Center, University of North Carolina at Chapel Hill, Chapel Hill, NC USA; 545 University of Tennessee Health Science Center for Cancer Research, Memphis, TN USA; 546 Department of Histopathology, Salford Royal NHS Foundation Trust, Salford, UK; 547 Faculty of Biology, Medicine and Health, University of Manchester, Manchester, UK; 548 BIOPIC, ICG and College of Life Sciences, Peking University, Beijing, China; 549 Peking-Tsinghua Center for Life Sciences, Peking University, Beijing, China; 550 Children’s Hospital of Philadelphia, Philadelphia, PA USA; 551 Department of Bioinformatics and Computational Biology and Department of Systems Biology, The University of Texas MD Anderson Cancer Center, Houston, TX USA; 552 Karolinska Institute, Stockholm, Sweden; 553 The Donnelly Centre, University of Toronto, Toronto, ON Canada; 554 Department of Medical Genetics, College of Medicine, Hallym University, Chuncheon, South Korea; 555 Department of Experimental and Health Sciences, Institute of Evolutionary Biology (UPF-CSIC), Universitat Pompeu Fabra, Barcelona, Spain; 556 Health Data Science Unit, University Clinics, Heidelberg, Germany; 557 Massachusetts General Hospital Center for Cancer Research, Charlestown, MA USA; 558 Hokkaido University, Sapporo, Japan; 559 Department of Pathology and Clinical Laboratory, National Cancer Center Hospital, Tokyo, Japan; 560 Department of Genetics, University of North Carolina at Chapel Hill, Chapel Hill, NC USA; 561 Computational Biology, Leibniz Institute on Aging - Fritz Lipmann Institute (FLI), Jena, Germany; 562 University of Melbourne Centre for Cancer Research, Melbourne, VIC Australia; 563 University of Nebraska Medical Center, Omaha, NE USA; 564 Syntekabio Inc, Daejeon, South Korea; 565 Department of Pathology, Academic Medical Center, Amsterdam, AZ The Netherlands; 566 China National GeneBank-Shenzhen, Shenzhen, China; 567 Division of Molecular Genetics, German Cancer Research Center (DKFZ), Heidelberg, Germany; 568 Division of Life Science and Applied Genomics Center, Hong Kong University of Science and Technology, Clear Water Bay, Hong Kong, China; 569 Icahn School of Medicine at Mount Sinai, New York, NY USA; 570 Geneplus-Shenzhen, Shenzhen, China; 571 School of Computer Science and Technology, Xi’an Jiaotong University, Xi’an, China; 572 AbbVie, North Chicago, IL USA; 573 Institute of Pathology, Charité – University Medicine Berlin, Berlin, Germany; 574 Centre for Translational and Applied Genomics, British Columbia Cancer Agency, Vancouver, BC Canada; 575 Edinburgh Royal Infirmary, Edinburgh, UK; 576 Berlin Institute for Medical Systems Biology, Max Delbrück Center for Molecular Medicine, Berlin, Germany; 577 Department of Pediatric Immunology, Hematology and Oncology, University Hospital, Heidelberg, Germany; 578 German Cancer Research Center (DKFZ), Heidelberg, Germany; 579 Heidelberg Institute for Stem Cell Technology and Experimental Medicine (HI-STEM), Heidelberg, Germany; 580 Institute for Computational Biomedicine, Weill Cornell Medical College, New York, NY USA; 581 New York Genome Center, New York, NY USA; 582 Department of Urology, James Buchanan Brady Urological Institute, Johns Hopkins University School of Medicine, Baltimore, MD USA; 583 Department of Preventive Medicine, Graduate School of Medicine, The University of Tokyo, Tokyo, Japan; 584 Department of Molecular and Cellular Biology, Baylor College of Medicine, Houston, TX USA; 585 Department of Pathology and Immunology, Baylor College of Medicine, Houston, TX USA; 586 Michael E. DeBakey Veterans Affairs Medical Center, Houston, TX USA; 587 Technical University of Denmark, Lyngby, Denmark; 588 Department of Pathology, College of Medicine, Hanyang University, Seoul, South Korea; 589 Academic Unit of Surgery, School of Medicine, College of Medical, Veterinary and Life Sciences, University of Glasgow, Glasgow Royal Infirmary, Glasgow, UK; 590 Department of Pathology, Asan Medical Center, College of Medicine, Ulsan University, Songpa-gu, Seoul South Korea; 591 Science Writer, Garrett Park, MD USA; 592 International Cancer Genome Consortium (ICGC)/ICGC Accelerating Research in Genomic Oncology (ARGO) Secretariat, Ontario Institute for Cancer Research, Toronto, ON Canada; 593 University of Ljubljana, Ljubljana, Slovenia; 594 Department of Public Health Sciences, University of Chicago, Chicago, IL USA; 595 Research Institute, NorthShore University HealthSystem, Evanston, IL USA; 596 Department for Biomedical Research, University of Bern, Bern, Switzerland; 597 Centre of Genomics and Policy, McGill University and Génome Québec Innovation Centre, Montreal, QC Canada; 598 Carolina Center for Genome Sciences, University of North Carolina at Chapel Hill, Chapel Hill, NC USA; 599 Hopp Children’s Cancer Center (KiTZ), Heidelberg, Germany; 600 Pediatric Glioma Research Group, German Cancer Research Center (DKFZ), Heidelberg, Germany; 601 Cancer Research UK, London, UK; 602 Indivumed GmbH, Hamburg, Germany; 603 Genome Integration Data Center, Syntekabio, Inc, Daejeon, South Korea; 604 University Hospital Zurich, Zurich, Switzerland; 605 Clinical Bioinformatics, Swiss Institute of Bioinformatics, Geneva, Switzerland; 606 Institute for Pathology and Molecular Pathology, University Hospital Zurich, Zurich, Switzerland; 607 Institute of Molecular Life Sciences, University of Zurich, Zurich, Switzerland; 608 MRC Human Genetics Unit, MRC IGMM, University of Edinburgh, Edinburgh, UK; 609 Women’s Cancer Program at the Samuel Oschin Comprehensive Cancer Institute, Cedars-Sinai Medical Center, Los Angeles, CA USA; 610 Department of Biology, Bioinformatics Group, Division of Molecular Biology, Faculty of Science, University of Zagreb, Zagreb, Croatia; 611 Department for Internal Medicine II, University Hospital Schleswig-Holstein, Kiel, Germany; 612 Genetics and Molecular Pathology, SA Pathology, Adelaide, SA Australia; 613 Department of Gastric Surgery, National Cancer Center Hospital, Tokyo, Japan; 614 Department of Bioinformatics, Division of Cancer Genomics, National Cancer Center Research Institute, Tokyo, Japan; 615 A.A. Kharkevich Institute of Information Transmission Problems, Moscow, Russia; 616 Oncology and Immunology, Dmitry Rogachev National Research Center of Pediatric Hematology, Moscow, Russia; 617 Skolkovo Institute of Science and Technology, Moscow, Russia; 618 Department of Surgery, The George Washington University, School of Medicine and Health Science, Washington, DC USA; 619 Endocrine Oncology Branch, Center for Cancer Research, National Cancer Institute, National Institutes of Health, Bethesda, MD USA; 620 Melanoma Institute Australia, Macquarie University, Sydney, NSW Australia; 621 MIT Computer Science and Artificial Intelligence Laboratory, Massachusetts Institute of Technology, Cambridge, MA USA; 622 Tissue Pathology and Diagnostic Oncology, Royal Prince Alfred Hospital, Sydney, NSW Australia; 623 Cholangiocarcinoma Screening and Care Program and Liver Fluke and Cholangiocarcinoma Research Centre, Faculty of Medicine, Khon Kaen University, Khon Kaen, Thailand; 624 Controlled Department and Institution, New York, NY USA; 625 Englander Institute for Precision Medicine, Weill Cornell Medicine, New York, NY USA; 626 National Cancer Center, Gyeonggi, South Korea; 627 Department of Biochemistry, College of Medicine, Ewha Womans University, Seoul, South Korea; 628 Health Sciences Department of Biomedical Informatics, University of California San Diego, La Jolla, CA USA; 629 Research Core Center, National Cancer Centre Korea, Goyang-si, South Korea; 630 Department of Health Sciences and Technology, Sungkyunkwan University School of Medicine, Seoul, South Korea; 631 Samsung Genome Institute, Seoul, South Korea; 632 Breast Oncology Program, Dana-Farber/Brigham and Women’s Cancer Center, Boston, MA USA; 633 Department of Surgery, Memorial Sloan Kettering Cancer Center, New York, NY USA; 634 Division of Breast Surgery, Brigham and Women’s Hospital, Boston, MA USA; 635 Integrative Bioinformatics Support Group, National Institute of Environmental Health Sciences (NIEHS), Durham, NC USA; 636 Department of Clinical Science, University of Bergen, Bergen, Norway; 637 Center For Medical Innovation, Seoul National University Hospital, Seoul, South Korea; 638 Department of Internal Medicine, Seoul National University Hospital, Seoul, South Korea; 639 Institute of Computer Science, Polish Academy of Sciences, Warsawa, Poland; 640 Functional and Structural Genomics, German Cancer Research Center (DKFZ), Heidelberg, Germany; 641 Laboratory of Translational Genomics, Division of Cancer Epidemiology and Genetics, National Cancer Institute, , National Institutes of Health, Bethesda, MD USA; 642 Institute for Medical Informatics Statistics and Epidemiology, University of Leipzig, Leipzig, Germany; 643 Morgan Welch Inflammatory Breast Cancer Research Program and Clinic, The University of Texas MD Anderson Cancer Center, Houston, TX USA; 644 Department of Hematology and Oncology, Georg-Augusts-University of Göttingen, Göttingen, Germany; 645 Institute of Cell Biology (Cancer Research), University of Duisburg-Essen, Essen, Germany; 646 King’s College London and Guy’s and St. Thomas’ NHS Foundation Trust, London, UK; 647 Center for Epigenetics, Van Andel Research Institute, Grand Rapids, MI USA; 648 The University of Queensland Centre for Clinical Research, Royal Brisbane and Women’s Hospital, Herston, QLD Australia; 649 Department of Pediatric Oncology and Hematology, University of Cologne, Cologne, Germany; 650 University of Düsseldorf, Düsseldorf, Germany; 651 Department of Pathology, Institut Jules Bordet, Brussels, Belgium; 652 Institute of Biomedicine, Sahlgrenska Academy at University of Gothenburg, Gothenburg, Sweden; 653 Children’s Medical Research Institute, Sydney, NSW Australia; 654 ILSbio, LLC Biobank, Chestertown, MD USA; 655 Division of Genetics and Genomics, Boston Children’s Hospital, Harvard Medical School, Boston, MA USA; 656 Institute for Bioengineering and Biopharmaceutical Research (IBBR), Hanyang University, Seoul, South Korea; 657 Department of Statistics, University of California Santa Cruz, Santa Cruz, CA USA; 658 National Genotyping Center, Institute of Biomedical Sciences, Academia Sinica, Taipei, Taiwan; 659 Department of Vertebrate Genomics/Otto Warburg Laboratory Gene Regulation and Systems Biology of Cancer, Max Planck Institute for Molecular Genetics, Berlin, Germany; 660 McGill University and Genome Quebec Innovation Centre, Montreal, QC Canada; 661 biobyte solutions GmbH, Heidelberg, Germany; 662 Gynecologic Oncology, NYU Laura and Isaac Perlmutter Cancer Center, New York University, New York, NY USA; 663 Division of Oncology, Stem Cell Biology Section, Washington University School of Medicine, St. Louis, MO USA; 664 Department of Systems Biology, The University of Texas MD Anderson Cancer Center, Houston, TX USA; 665 Harvard University, Cambridge, MA USA; 666 Urologic Oncology Branch, Center for Cancer Research, National Cancer Institute, National Institutes of Health, Bethesda, MD USA; 667 University of Oslo, Oslo, Norway; 668 University of Toronto, Toronto, ON Canada; 669 Peking University, Beijing, China; 670 School of Life Sciences, Peking University, Beijing, China; 671 Leidos Biomedical Research, Inc, McLean, VA USA; 672 Hematology, Hospital Clinic, Institut d’Investigacions Biomèdiques August Pi i Sunyer (IDIBAPS), University of Barcelona, Barcelona, Spain; 673 Second Military Medical University, Shanghai, China; 674 Chinese Cancer Genome Consortium, Shenzhen, China; 675 Department of Medical Oncology, Beijing Hospital, Beijing, China; 676 Laboratory of Molecular Oncology, Key Laboratory of Carcinogenesis and Translational Research (Ministry of Education), Peking University Cancer Hospital and Institute, Beijing, China; 677 School of Medicine/School of Mathematics and Statistics, University of St. Andrews, St, Andrews, Fife UK; 678 Institute for Systems Biology, Seattle, WA USA; 679 Department of Biochemistry and Molecular Biology, Faculty of Medicine, University Institute of Oncology-IUOPA, Oviedo, Spain; 680 Institut Bergonié, Bordeaux, France; 681 Cancer Unit, MRC University of Cambridge, Cambridge, UK; 682 Department of Pathology and Laboratory Medicine, Center for Personalized Medicine, Children’s Hospital Los Angeles, Los Angeles, CA USA; 683 John Curtin School of Medical Research, Canberra, ACT Australia; 684 MVZ Department of Oncology, PraxisClinic am Johannisplatz, Leipzig, Germany; 685 Department of Information Technology, Ghent University, Ghent, Belgium; 686 Department of Plant Biotechnology and Bioinformatics, Ghent University, Ghent, Belgium; 687 Institute for Genomic Medicine, Nationwide Children’s Hospital, Columbus, OH USA; 688 Computational Biology Program, School of Medicine, Oregon Health and Science University, Portland, OR USA; 689 Department of Surgery, Duke University, Durham, NC USA; 690 Institució Catalana de Recerca i Estudis Avançats (ICREA), Barcelona, Spain; 691 Institut Català de Paleontologia Miquel Crusafont, Universitat Autònoma de Barcelona, Barcelona, Spain; 692 University of Glasgow, Glasgow, UK; 693 Institut d’Investigacions Biomèdiques August Pi i Sunyer (IDIBAPS), Barcelona, Spain; 694 Division of Oncology, Washington University School of Medicine, St. Louis, MO USA; 695 Department of Surgery and Cancer, Imperial College, London, INY UK; 696 Applications Department, Oxford Nanopore Technologies, Oxford, UK; 697 Department of Obstetrics, Gynecology and Reproductive Services, University of California San Francisco, San Francisco, CA USA; 698 Department of Biochemistry and Molecular Medicine, University California at Davis, Sacramento, CA USA; 699 STTARR Innovation Facility, Princess Margaret Cancer Centre, Toronto, ON Canada; 700 Discipline of Surgery, Western Sydney University, Penrith, NSW Australia; 701 Yale School of Medicine, Yale University, New Haven, CT USA; 702 Department of Genetics, Lineberger Comprehensive Cancer Center, University of North Carolina at Chapel Hill, Chapel Hill, NC USA; 703 Departments of Neurology and Neurosurgery, Henry Ford Hospital, Detroit, MI USA; 704 Precision Oncology, OHSU Knight Cancer Institute, Oregon Health and Science University, Portland, OR USA; 705 Institute of Pathology, University Medical Center Hamburg-Eppendorf, Hamburg, Germany; 706 Department of Health Sciences, Faculty of Medical Sciences, Kyushu University, Fukuoka, Japan; 707 Heidelberg Academy of Sciences and Humanities, Heidelberg, Germany; 708 Department of Clinical Pathology, University of Melbourne, Melbourne, VIC, Australia; 709 Department of Pathology, Roswell Park Cancer Institute, Buffalo, NY USA; 710 Department of Computer Science, University of Helsinki, Helsinki, Finland; 711 Institute of Biotechnology, University of Helsinki, Helsinki, Finland; 712 Organismal and Evolutionary Biology Research Programme, University of Helsinki, Helsinki, Finland; 713 Department of Obstetrics and Gynecology, Division of Gynecologic Oncology, Washington University School of Medicine, St. Louis, MO USA; 714 Penrose St. Francis Health Services, Colorado Springs, CO USA; 715 Institute of Pathology, Ulm University and University Hospital of Ulm, Ulm, Germany; 716 National Cancer Center, Tokyo, Japan; 717 Genome Institute of Singapore, Singapore, Singapore; 718 32Program in Computational Biology and Bioinformatics, Yale University, New Haven, CT USA; 719 German Cancer Aid, Bonn, Germany; 720 Programme in Cancer and Stem Cell Biology, Centre for Computational Biology, Duke-NUS Medical School, Singapore, Singapore; 721 The Chinese University of Hong Kong, Shatin, NT, Hong Kong China; 722 Fourth Military Medical University, Shaanxi, China; 723 The University of Cambridge School of Clinical Medicine, Cambridge, UK; 724 St. Jude Children’s Research Hospital, Memphis, TN USA; 725 University Health Network, Princess Margaret Cancer Centre, Toronto, ON Canada; 726 Center for Biomolecular Science and Engineering, University of California Santa Cruz, Santa Cruz, CA USA; 727 Department of Medicine, University of Chicago, Chicago, IL USA; 728 Department of Neurology, Mayo Clinic, Rochester, MN USA; 729 Cambridge Oesophagogastric Centre, Cambridge University Hospitals NHS Foundation Trust, Cambridge, UK; 730 Department of Computer Science, Carleton College, Northfield, MN USA; 731 Institute of Cancer Sciences, College of Medical Veterinary and Life Sciences, University of Glasgow, Glasgow, UK; 732 Department of Epidemiology, University of Alabama at Birmingham, Birmingham, AL USA; 733 HudsonAlpha Institute for Biotechnology, Huntsville, AL USA; 734 O’Neal Comprehensive Cancer Center, University of Alabama at Birmingham, Birmingham, AL USA; 735 Department of Pathology, Keio University School of Medicine, Tokyo, Japan; 736 Department of Hepatobiliary and Pancreatic Oncology, National Cancer Center Hospital, Tokyo, Japan; 737 Sage Bionetworks, Seattle, WA USA; 738 Lymphoma Genomic Translational Research Laboratory, National Cancer Centre, Singapore, Singapore; 739 Department of Clinical Pathology, Robert-Bosch-Hospital, Stuttgart, Germany; 740 Department of Cell and Systems Biology, University of Toronto, Toronto, ON Canada; 741 Department of Biosciences and Nutrition, Karolinska Institutet, Stockholm, Sweden; 742 Center for Liver Cancer, Research Institute and Hospital, National Cancer Center, Gyeonggi, South Korea; 743 Division of Hematology-Oncology, Samsung Medical Center, Sungkyunkwan University School of Medicine, Seoul, South Korea; 744 Samsung Advanced Institute for Health Sciences and Technology, Sungkyunkwan University School of Medicine, Seoul, South Korea; 745 Cheonan Industry-Academic Collaboration Foundation, Sangmyung University, Cheonan, South Korea; 746 NYU Langone Medical Center, New York, NY USA; 747 Department of Hematology and Medical Oncology, Cleveland Clinic, Cleveland, OH USA; 748 Department of Radiation Oncology, University of California San Francisco, San Francisco, CA USA; 749 Department of Health Sciences Research, Mayo Clinic, Rochester, MN USA; 750 Helen F. Graham Cancer Center at Christiana Care Health Systems, Newark, DE USA; 751 Heidelberg University Hospital, Heidelberg, Germany; 752 CSRA Incorporated, Fairfax, VA USA; 753 Research Department of Pathology, University College London Cancer Institute, London, UK; 754 Department of Research Oncology, Guy’s Hospital, King’s Health Partners AHSC, King’s College London School of Medicine, London, UK; 755 Faculty of Medicine and Health Sciences, Macquarie University, Sydney, NSW Australia; 756 University Hospital of Minjoz, INSERM UMR 1098, Besançon, France; 757 Spanish National Cancer Research Centre, Madrid, Spain; 758 Center of Digestive Diseases and Liver Transplantation, Fundeni Clinical Institute, Bucharest, Romania; 759 Cureline, Inc, South San Francisco, CA USA; 760 St. Luke’s Cancer Centre, Royal Surrey County Hospital NHS Foundation Trust, Guildford, UK; 761 Cambridge Breast Unit, Addenbrooke’s Hospital, Cambridge University Hospital NHS Foundation Trust and NIHR Cambridge Biomedical Research Centre, Cambridge, UK; 762 East of Scotland Breast Service, Ninewells Hospital, Aberdeen, UK; 763 Department of Genetics, Microbiology and Statistics, University of Barcelona, IRSJD, IBUB, Barcelona, Spain; 764 Department of Obstetrics and Gynecology, Medical College of Wisconsin, Milwaukee, WI USA; 765 Hematology and Medical Oncology, Winship Cancer Institute of Emory University, Atlanta, GA USA; 766 Department of Computer Science, Princeton University, Princeton, NJ USA; 767 Vanderbilt Ingram Cancer Center, Vanderbilt University, Nashville, TN USA; 768 Ohio State University College of Medicine and Arthur G. James Comprehensive Cancer Center, Columbus, OH USA; 769 Department of Surgery, Yokohama City University Graduate School of Medicine, Kanagawa, Japan; 770 Division of Chromatin Networks, German Cancer Research Center (DKFZ) and BioQuant, Heidelberg, Germany; 771 Research Computing Center, University of North Carolina at Chapel Hill, Chapel Hill, NC USA; 772 School of Molecular Biosciences and Center for Reproductive Biology, Washington State University, Pullman, WA USA; 773 Finsen Laboratory and Biotech Research and Innovation Centre (BRIC), University of Copenhagen, Copenhagen, Denmark; 774 Department of Laboratory Medicine and Pathobiology, University of Toronto, Toronto, ON Canada; 775 Department of Pathology, Human Oncology and Pathogenesis Program, Memorial Sloan Kettering Cancer Center, New York, NY USA; 776 University Hospital Giessen, Pediatric Hematology and Oncology, Giessen, Germany; 777 Oncologie Sénologie, ICM Institut Régional du Cancer, Montpellier, France; 778 Institute of Clinical Molecular Biology, Christian-Albrechts-University, Kiel, Germany; 779 Institute of Pathology, University of Wuerzburg, Wuerzburg, Germany; 780 Department of Urology, North Bristol NHS Trust, Bristol, UK; 781 SingHealth, Duke-NUS Institute of Precision Medicine, National Heart Centre Singapore, Singapore, Singapore; 782 Department of Computer Science, University of Toronto, Toronto, ON Canada; 783 Bern Center for Precision Medicine, University Hospital of Bern, University of Bern, Bern, Switzerland; 784 Englander Institute for Precision Medicine, Weill Cornell Medicine and New York Presbyterian Hospital, New York, NY USA; 785 Meyer Cancer Center, Weill Cornell Medicine, New York, NY USA; 786 Pathology and Laboratory, Weill Cornell Medical College, New York, NY USA; 787 Vall d’Hebron Institute of Oncology: VHIO, Barcelona, Spain; 788 General and Hepatobiliary-Biliary Surgery, Pancreas Institute, University and Hospital Trust of Verona, Verona, Italy; 789 National Centre for Biological Sciences, Tata Institute of Fundamental Research, Bangalore, India; 790 Indiana University, Bloomington, IN USA; 791 Department of Pathology, GZA-ZNA Hospitals, Antwerp, Belgium; 792 Analytical Biological Services, Inc, Wilmington, DE USA; 793 Sydney Medical School, University of Sydney, Sydney, NSW Australia; 794 cBio Center, Dana-Farber Cancer Institute, Harvard Medical School, Boston, MA USA; 795 Department of Cell Biology, Harvard Medical School, Boston, MA USA; 796 Advanced Centre for Treatment Research and Education in Cancer, Tata Memorial Centre, Navi Mumbai, Maharashtra India; 797 School of Environmental and Life Sciences, Faculty of Science, The University of Newcastle, Ourimbah, NSW Australia; 798 Department of Dermatology, University Hospital of Essen, Essen, Germany; 799 Bioinformatics and Omics Data Analytics, German Cancer Research Center (DKFZ), Heidelberg, Germany; 800 Department of Urology, Charité Universitätsmedizin Berlin, Berlin, Germany; 801 Martini-Clinic, Prostate Cancer Center, University Medical Center Hamburg-Eppendorf, Hamburg, Germany; 802 Department of General Internal Medicine, University of Kiel, Kiel, Germany; 803 German Cancer Consortium (DKTK), Partner site Berlin, Berlin, Germany; 804 Cancer Research Institute, Beth Israel Deaconess Medical Center, Boston, MA USA; 805 University of Pittsburgh, Pittsburgh, PA USA; 806 Department of Ophthalmology and Ocular Genomics Institute, Massachusetts Eye and Ear, Harvard Medical School, Boston, MA USA; 807 Center for Psychiatric Genetics, NorthShore University HealthSystem, Evanston, IL USA; 808 Van Andel Research Institute, Grand Rapids, MI USA; 809 Laboratory of Molecular Medicine, Human Genome Center, Institute of Medical Science, University of Tokyo, Tokyo, Japan; 810 Japan Agency for Medical Research and Development, Tokyo, Japan; 811 Korea University, Seoul, South Korea; 812 Murtha Cancer Center, Walter Reed National Military Medical Center, Bethesda, MD USA; 813 Human Genetics, University of Kiel, Kiel, Germany; 814 Department of Oncologic Pathology, Dana-Farber Cancer Institute, Harvard Medical School, Boston, MA USA; 815 Oregon Health and Science University, Portland, OR USA; 816 Center for RNA Interference and Noncoding RNA, The University of Texas MD Anderson Cancer Center, Houston, TX USA; 817 Department of Experimental Therapeutics, The University of Texas MD Anderson Cancer Center, Houston, TX USA; 818 Department of Gynecologic Oncology and Reproductive Medicine, The University of Texas MD Anderson Cancer Center, Houston, TX USA; 819 University Hospitals Coventry and Warwickshire NHS Trust, Coventry, UK; 820 Department of Radiation Oncology, Radboud University Nijmegen Medical Centre, Nijmegen, GA The Netherlands; 821 Institute for Genomics and Systems Biology, University of Chicago, Chicago, IL USA; 822 Clinic for Hematology and Oncology, St.-Antonius-Hospital, Eschweiler, Germany; 823 Computational and Systems Biology Program, Memorial Sloan Kettering Cancer Center, New York, NY USA; 824 University of Iceland, Reykjavik, Iceland; 825 Division of Computational Genomics and Systems Genetics, German Cancer Research Center (DKFZ), Heidelberg, Germany; 826 Dundee Cancer Centre, Ninewells Hospital, Dundee, UK; 827 Department for Internal Medicine III, University of Ulm and University Hospital of Ulm, Ulm, Germany; 828 Institut Curie, INSERM Unit 830, Paris, France; 829 Department of Gastroenterology and Hepatology, Yokohama City University Graduate School of Medicine, Kanagawa, Japan; 830 Department of Laboratory Medicine, Radboud University Nijmegen Medical Centre, Nijmegen, GA The Netherlands; 831 Division of Cancer Genome Research, German Cancer Research Center (DKFZ), Heidelberg, Germany; 832 Department of General Surgery, Singapore General Hospital, Singapore, Singapore; 833 Cancer Science Institute of Singapore, National University of Singapore, Singapore, Singapore; 834 Department of Medical and Clinical Genetics, Genome-Scale Biology Research Program, University of Helsinki, Helsinki, Finland; 835 East Anglian Medical Genetics Service, Cambridge University Hospitals NHS Foundation Trust, Cambridge, UK; 836 Irving Institute for Cancer Dynamics, Columbia University, New York, NY USA; 837 Institute of Molecular and Cell Biology, Singapore, Singapore; 838 Laboratory of Cancer Epigenome, Division of Medical Science, National Cancer Centre Singapore, Singapore, Singapore; 839 Universite Lyon, INCa-Synergie, Centre Léon Bérard, Lyon, France; 840 Department of Urology, Mayo Clinic, Rochester, MN USA; 841 Royal National Orthopaedic Hospital - Stanmore, Stanmore, Middlesex UK; 842 Department of Biochemistry, Genetics and Immunology, University of Vigo, Vigo, Spain; 843 Giovanni Paolo II / I.R.C.C.S. Cancer Institute, Bari, BA Italy; 844 Neuroblastoma Genomics, German Cancer Research Center (DKFZ), Heidelberg, Germany; 845 Fondazione Policlinico Universitario Gemelli IRCCS, Rome, Italy, Rome, Italy; 846 University of Verona, Verona, Italy; 847 Centre National de Génotypage, CEA - Institute de Génomique, Evry, France; 848 CAPHRI Research School, Maastricht University, Maastricht, ER The Netherlands; 849 Department of Biopathology, Centre Léon Bérard, Lyon, France; 850 Université Claude Bernard Lyon 1, Villeurbanne, France; 851 Core Research for Evolutional Science and Technology (CREST), JST, Tokyo, Japan; 852 Department of Biological Sciences, Laboratory for Medical Science Mathematics, Graduate School of Science, University of Tokyo, Yokohama, Japan; 853 Department of Medical Science Mathematics, Medical Research Institute, Tokyo Medical and Dental University (TMDU), Tokyo, Japan; 854 Cancer Ageing and Somatic Mutation Programme, Wellcome Sanger Institute, Hinxton, UK; 855 University Hospitals Birmingham NHS Foundation Trust, Birmingham, UK; 856 Centre for Cancer Research and Cell Biology, Queen’s University, Belfast, UK; 857 Breast Medical Oncology, The University of Texas MD Anderson Cancer Center, Houston, TX USA; 858 Department of Surgery, Johns Hopkins University School of Medicine, Baltimore, MD USA; 859 Department of Oncology-Pathology, Science for Life Laboratory, Karolinska Institute, Stockholm, Sweden; 860 School of Cancer Sciences, Faculty of Medicine, University of Southampton, Southampton, UK; 861 Department of Gene Technology, Tallinn University of Technology, Tallinn, Estonia; 862 Genetics and Genome Biology Program, SickKids Research Institute, The Hospital for Sick Children, Toronto, ON Canada; 863 Departments of Neurosurgery and Hematology and Medical Oncology, Winship Cancer Institute and School of Medicine, Emory University, Atlanta, GA USA; 864 Department of Clinical and Molecular Medicine, Faculty of Medicine and Health Sciences, Norwegian University of Science and Technology, Trondheim, Norway; 865 Argmix Consulting, North Vancouver, BC Canada; 866 Department of Information Technology, Ghent University, Interuniversitair Micro-Electronica Centrum (IMEC), Ghent, Belgium; 867 Nuffield Department of Surgical Sciences, John Radcliffe Hospital, University of Oxford, Oxford, UK; 868 Institute of Mathematics and Computer Science, University of Latvia, Riga, LV Latvia; 869 Discipline of Pathology, Sydney Medical School, University of Sydney, Sydney, NSW Australia; 870 Department of Applied Mathematics and Theoretical Physics, Centre for Mathematical Sciences, University of Cambridge, Cambridge, UK; 871 Department of Epidemiology and Biostatistics, Memorial Sloan Kettering Cancer Center, New York, NY USA; 872 Department of Statistics, Columbia University, New York, NY USA; 873 Department of Immunology, Genetics and Pathology, Science for Life Laboratory, Uppsala University, Uppsala, Sweden; 874 School of Electronic and Information Engineering, Xi’an Jiaotong University, Xi’an, China; 875 Department of Histopathology, Cambridge University Hospitals NHS Foundation Trust, Cambridge, UK; 876 Oxford NIHR Biomedical Research Centre, University of Oxford, Oxford, UK; 877 Georgia Regents University Cancer Center, Augusta, GA USA; 878 Wythenshawe Hospital, Manchester, UK; 879 Department of Genetics, Washington University School of Medicine, St.Louis, MO USA; 880 Department of Biological Oceanography, Leibniz Institute of Baltic Sea Research, Rostock, Germany; 881 Wellcome Centre for Human Genetics, University of Oxford, Oxford, UK; 882 Department of Molecular and Human Genetics, Baylor College of Medicine, Houston, TX USA; 883 Thoracic Oncology Laboratory, Mayo Clinic, Rochester, MN USA; 884 Institute for Genomic Medicine, Nationwide Children’s Hospital, Columbus, OH USA; 885 Department of Obstetrics and Gynecology, Division of Gynecologic Oncology, Mayo Clinic, Rochester, MN USA; 886 International Institute for Molecular Oncology, Poznań, Poland; 887 Poznan University of Medical Sciences, Poznań, Poland; 888 Genomics and Proteomics Core Facility High Throughput Sequencing Unit, German Cancer Research Center (DKFZ), Heidelberg, Germany; 889 NCCS-VARI Translational Research Laboratory, National Cancer Centre Singapore, Singapore, Singapore; 890 Edison Family Center for Genome Sciences and Systems Biology, Washington University, St. Louis, MO USA; 891 MRC-University of Glasgow Centre for Virus Research, Glasgow, UK; 892 Department of Medical Informatics and Clinical Epidemiology, Division of Bioinformatics and Computational Biology, OHSU Knight Cancer Institute, Oregon Health and Science University, Portland, OR USA; 893 School of Electronic Information and Communications, Huazhong University of Science and Technology, Wuhan, China; 894 Department of Applied Mathematics and Statistics, Johns Hopkins University, Baltimore, MD USA; 895 Department of Cancer Genome Informatics, Graduate School of Medicine, Osaka University, Osaka, Japan; 896 Institute of Computer Science, Heidelberg University, Heidelberg, Germany; 897 School of Mathematics and Statistics, University of Sydney, Sydney, NSW Australia; 898 Ben May Department for Cancer Research, University of Chicago, Chicago, IL USA; 899 Department of Human Genetics, University of Chicago, Chicago, IL USA; 900 Tri-Institutional PhD Program in Computational Biology and Medicine, Weill Cornell Medicine, New York, NY USA; 901 The First Affiliated Hospital, Xi’an Jiaotong University, Xi’an, China; 902 Department of Medicine and Therapeutics, The Chinese University of Hong Kong, Shatin, NT, Hong Kong China; 903 Department of Biostatistics, The University of Texas MD Anderson Cancer Center, Houston, TX USA; 904 Duke-NUS Medical School, Singapore, Singapore; 905 Department of Surgery, Ruijin Hospital, Shanghai Jiaotong University School of Medicine, Shanghai, China; 906 School of Computing Science, University of Glasgow, Glasgow, UK; 907 Division of Orthopaedic Surgery, Oslo University Hospital, Oslo, Norway; 908 Eastern Clinical School, Monash University, Melbourne, VIC Australia; 909 Epworth HealthCare, Richmond, VIC Australia; 910 Department of Biostatistics and Computational Biology, Dana-Farber Cancer Institute and Harvard Medical School, Boston, MA USA; 911 Department of Biomedical Informatics, College of Medicine, The Ohio State University, Columbus, OH USA; 912 The Ohio State University Comprehensive Cancer Center (OSUCCC – James), Columbus, OH USA; 913 The University of Texas School of Biomedical Informatics (SBMI) at Houston, Houston, TX USA; 914 Department of Biostatistics, University of North Carolina at Chapel Hill, Chapel Hill, NC USA; 915 Department of Biochemistry and Molecular Genetics, Feinberg School of Medicine, Northwestern University, Chicago, IL USA; 916 Faculty of Medicine and Health, University of Sydney, Sydney, NSW Australia; 917 Department of Pathology, Erasmus Medical Center Rotterdam, Rotterdam, GD The Netherlands; 918 Division of Molecular Carcinogenesis, The Netherlands Cancer Institute, Amsterdam, CX The Netherlands; 919 Institute of Molecular Life Sciences and Swiss Institute of Bioinformatics, University of Zurich, Zurich, Switzerland

**Keywords:** Data mining, Cancer, Gene expression

## Abstract

Chromatin is folded into successive layers to organize linear DNA. Genes within the same topologically associating domains (TADs) demonstrate similar expression and histone-modification profiles, and boundaries separating different domains have important roles in reinforcing the stability of these features. Indeed, domain disruptions in human cancers can lead to misregulation of gene expression. However, the frequency of domain disruptions in human cancers remains unclear. Here, as part of the Pan-Cancer Analysis of Whole Genomes (PCAWG) Consortium of the International Cancer Genome Consortium (ICGC) and The Cancer Genome Atlas (TCGA), which aggregated whole-genome sequencing data from 2,658 cancers across 38 tumor types, we analyzed 288,457 somatic structural variations (SVs) to understand the distributions and effects of SVs across TADs. Notably, SVs can lead to the fusion of discrete TADs, and complex rearrangements markedly change chromatin folding maps in the cancer genomes. Notably, only 14% of the boundary deletions resulted in a change in expression in nearby genes of more than twofold.

## Abstract (web summary)

A pan-cancer genomic analysis reports the effects of structural variations on chromatin domains (TADs). Most TAD disruptions do not result in appreciable changes in expression of nearby genes.

## Main

Genome organization inside the nucleus is hierarchically organized^1^. Chromosomes are organized into chromosome territories^2^. Inside chromosome territories, certain regions of the chromatin are attached to the nuclear periphery and form repressive nuclear lamin-associated domains (LADs)^3^. Recent chromosome conformation studies have revealed that mammalian chromosomes are structured into largely tissue-invariant TADs in which the DNA interactions are more frequent within a given domain than with regions in other domains^4,5^. TADs are considered to represent functional domains because a given TAD encompasses the regulatory elements for the genes inside the same domain^6,7^. Therefore, the integrity of the domain structures is important for the proper regulation of genes^8–12^. The disruption of domain boundaries can result in ectopic interactions between neighboring domains and affect the regulation of nearby genes^5,9^. Regulatory landscapes are an important part of human malignancies, and studies have shown that the ‘hijacking’ of enhancers can lead to overexpression of oncogenes (for example, growth factor independent 1 family oncogenes (*GFI1* and *GFI1B*)) in medulloblastoma^13^ or proto-oncogene *MECOM* activation due to an inversion between TADs in acute myeloid leukemia cells, which facilitates tumor formation^14^. Several other studies have reported the deregulation of chromatin folding structures in different cancer types^11,15,16^. Hence, genomic rearrangements can have a significant role in the reshuffling of TAD structures that results in altered gene regulation. Despite these recent examples of SVs that result in altered local enhancer–promoter landscapes, the frequency of such regulatory architecture rearrangements in cancer genomes remains unclear. Similarly, whether there are loci affected by potential changes in regulatory structure outside of those currently reported in the literature is unknown. To address these questions, we comprehensively characterized the effects of different SVs on TADs and gene-expression patterns observed in various tumor types to expand understanding of the link between chromatin folding and genomic rearrangements in cancer genomes.

## Results

### TAD boundaries are affected by different types of somatic SV in cancer genomes

**Extended Data Fig. 1:**
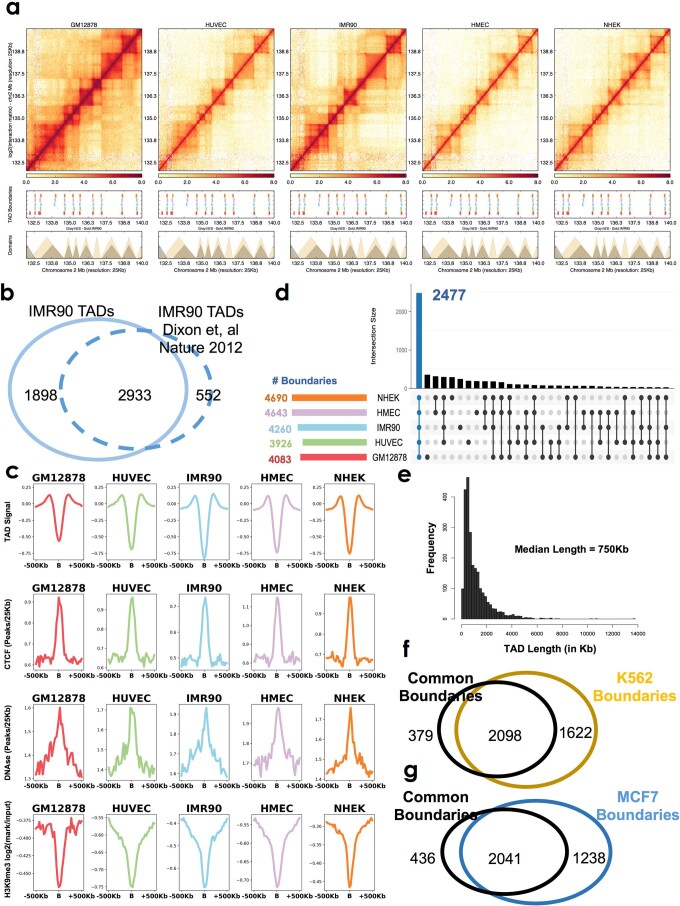
Identification of TAD boundaries in different cell types. **a**, An example region (chromosome2:132-140 Mb) presenting similar chromatin folding in 5 different cell types. Heatmaps represent Hi-C data for each cell type. Tiles represent TAD boundary calls for each cell type (red: GM12878; green: HUVEC; blue: IMR90; purple: HMEC; orange: NHEK). Triangles depict TAD calls for human ES cells (gray) and IMR90 cell line (gold) from a previous study^4^. **b**, Venn diagrams show overlap between current IMR90 boundaries (solid) with boundaries (dashed) identified from a previous study^4^ for the IMR90 cell line. **c**, Aggregate plots show average cell-type specific enrichment levels for Hi-C interaction levels (TAD signal), CTCF binding sites, DNAseI hypersensitivity regions and H3K9me3 ChIP-seq levels compared to input DNA around each cell type’s TAD boundaries. **d**, Overlaps between TAD boundaries among 5 different cell lines. Horizontal bars represent total number of TAD boundaries per cell type. Vertical bars represent number of intersecting boundaries between cell types. Combination matrix (below), circles indicate that denote cell types are part of the intersection for each vertical bars. Common boundaries among all cell types represented with blue vertical bar. **e**, Histogram represents distribution of TADs length. **f**, Venn diagrams show overlap between common TAD boundaries and leukemia (K562) cell line TAD boundaries. **g**, Venn diagrams show overlap between common TAD boundaries and breast cancer (MCF) cell line TAD boundaries.

Previous reports have indicated that TADs are a largely cell-type-invariant feature of genome organization^4,17^. In this pan-cancer analysis, we sought to generate a common set of boundaries observed in different cell types. We used high-resolution chromosome conformation (Hi-C) datasets from five human cell lines that represent three distinct embryonic germ layers (GM12878 and HMEC, mesoderm; IMR90, endoderm; HUVEC and NHEK, ectoderm)^17^ to identify TAD boundaries in different cell types (Extended Data Fig. 1a). We called TAD boundaries from 25-kb-binned Hi-C data for each cell type with an insulation score^18^ approach. This method calculates a score (TAD signal), for each bin, for the average interactions with the nearby loci for a 2-Mb genomic window. Boundaries are determined as regions with local insulation minima along the diagonal of the Hi-C matrix^18^. As a result, a number of boundaries, which ranged from 3,926 to 4,690, were found for different cell types. We next investigated whether our TAD boundary calls were consistent with the previously reported boundaries and showed attributes of TAD boundaries. To test this, we compared available boundary regions for IMR90 cells that were identified using a directionality-based approach (with a bin size of 40 kb)^4^. Our IMR90 boundary calls were highly overlapping (>84%) with published boundaries (Extended Data Fig. 1b). This showed that the current boundary regions were comparable with previously mapped boundaries even though they were identified at a different Hi-C resolution and using a different detection algorithm. Furthermore, we observed known TAD boundary signatures^4^ around our boundary calls for each cell type (Extended Data Fig. 1c). Across all cell types, we identified a common set of 2,477 boundaries (Supplementary Table 1, Extended Data Fig. 1d). There was a significant (*P* < 10^−6^) overlap (a 50-kb distance was allowed) between TAD boundaries among all profiled cell types. The median distance between the common boundaries was approximately 750 kb, consistent with the reported median TAD size in human cells^4,19^ (Extended Data Fig. 1e). The resulting 2,477 common regions were used for the rest of the analyses (referred to as boundaries hereafter).

**Fig. 1:**
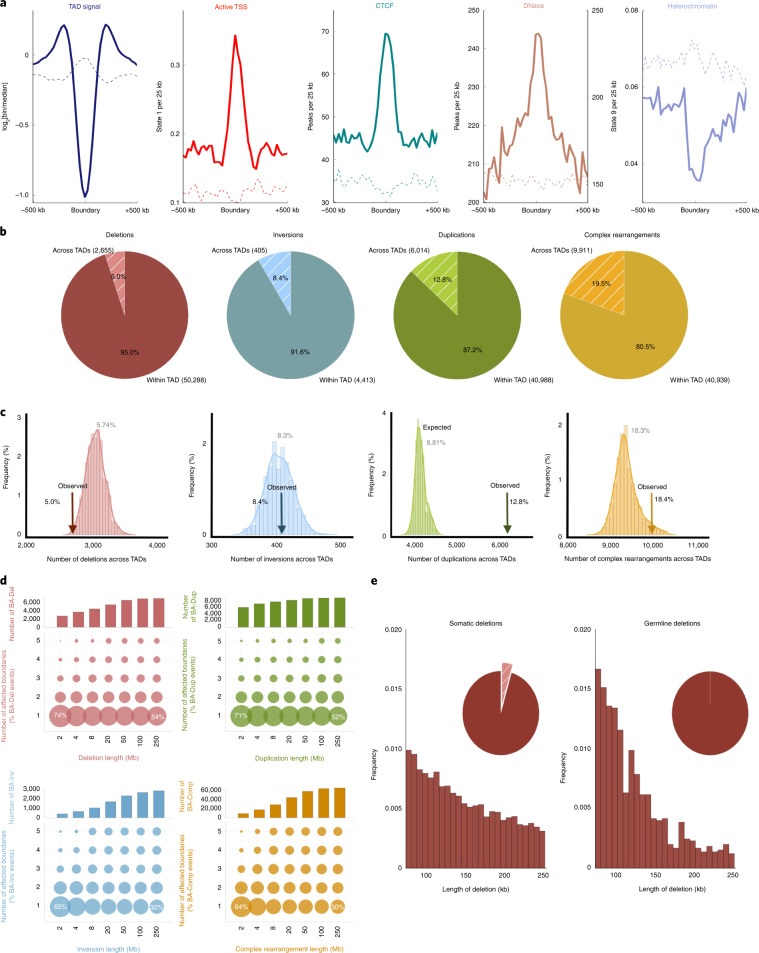
TAD boundaries are affected by different types of somatic SVs in cancer genomes. **a**, Profiles of TAD signals, active transcription start sites (TSS), CTCF peaks, DNase I hypersensitivity and heterochromatic states around common TAD boundaries. Dashed lines represent enrichment levels for the shuffled boundaries. **b**, The percentage of short-range SVs (length ≤ 2 Mb) across TADs (shaded) and within TADs (solid) for different SV types. SVs that occur across TADs are referred to as BA-SVs. **c**, Observed (arrows) and expected distribution (histograms) of BA-SVs. The expected distribution is based on randomly shuffled boundary data. **d**, Number of affected boundaries (*x* axis) per short-range SV length cut-off (*y* axis). The size of the circles indicates the portion of BA-SVs that affect the specific number of boundaries for each length scale. BA-deletion, BA-inversions, BA-duplications and BA-complex rearrangements are shown in red, cyan, green and orange, respectively. **e**, Histograms represent the length distribution of somatic and germline deletions in the PCAWG cohort within the 75–250 kb range. Pie charts show the percentage of deletions across TADs (shaded) (4.1% for somatic and less than 0.1% for germline events) and within TADs (solid).

Next, to test whether the overall chromatin architecture is similar in cancer and non-cancer cells, we intersected these boundaries with the TAD boundaries found in cancer cell lines. We observed a high overlap with boundaries from a leukemia cell line K562 (ref. ^17^) and a breast cancer cell line MCF7 (ref. ^20^) (85% and 83.4%, respectively; Extended Data Fig. 1f,g). These analyses revealed that a significant (*P* < 10^−7^) percentage of boundaries was conserved between normal and malignant cells. We next examined the enrichment of CCCTC-binding factor (CTCF)-binding and DNase I hypersensitivity sites, as well as active transcription start sites and heterochromatic regions around boundaries from various cell types that have previously been profiled by the Encyclopedia of DNA Elements (ENCODE) consortium^19^ and the Roadmap Epigenome project^21^. We observed that CTCF-binding sites and active promoter marks were enriched, whereas the heterochromatin state was depleted at the boundaries. In addition, TAD signal levels were the lowest at the boundaries compared with flanking sites (Fig. 1a), consistent with the role of TAD boundaries in the reduction of the contacts between adjacent domains. Overall, these common 2,477 boundaries exhibited the genomic features of TAD boundaries across different human cell types.

**Extended Data Fig. 2:**
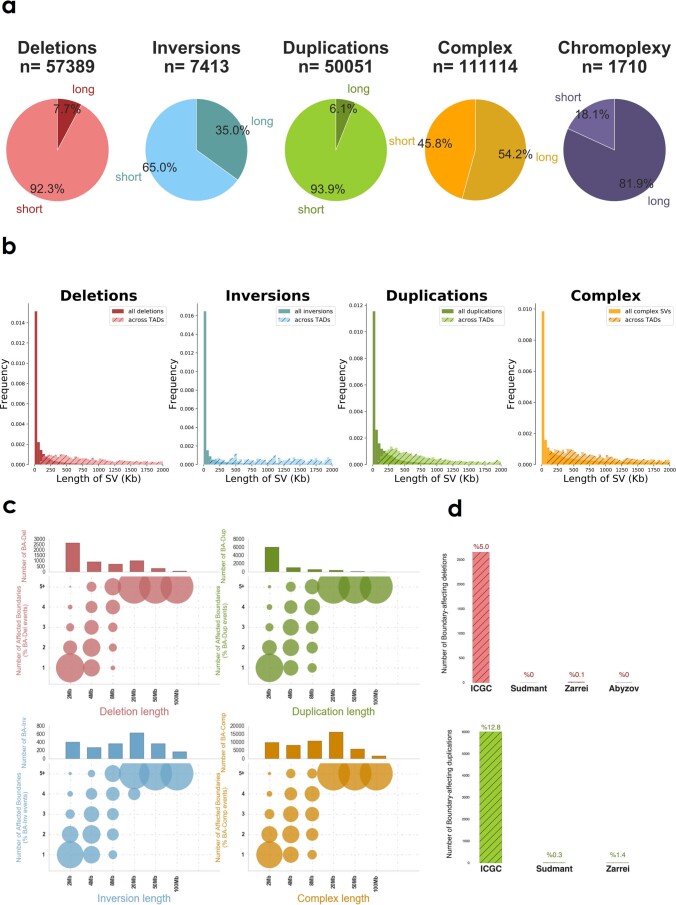
Distribution of boundary-affecting structural variations in human cancers. **a**, Pie charts show the percentages of long-range (>2 Mb) and short-range (< = 2 Mb) for deletions (red), inversions (cyan), duplications (green), complex rearrangements (orange) and chromoplexy events (purple) in all PCAWG samples. **b**, Histograms show length distribution of all short-range SVs (solid) or Boundary Affecting SVs (dashed) for deletions (red), inversions (cyan), duplications (green) and complex rearrangements (orange) in all PCAWG samples. **c**, Number of affected boundaries (x-axis) per different short-range SV length cut-offs (y-axis). The size of the circles indicates the portion of BA-SVs affecting the specific number of boundaries for each length scale. BA-deletion, BA-inversions, BA-duplications and BA-complex rearrangements are represented with red, cyan, green and orange colors, respectively. **d**, Bar charts show TAD-boundary affecting top) deletions (red) and bottom) tandem-duplications (green) in cancer genomes, and in genomes of healthy individuals from three different studies.

To understand the effects of SVs on TAD boundaries in human cancers, we used 288,457 high-confidence somatic SVs as part of the ICGC PCAWG project. The PCAWG Consortium aggregated whole-genome sequencing (WGS) data from 2,658 cancers across 38 tumor types generated by the ICGC and TCGA projects. These sequencing data were re-analyzed with standardized, high-accuracy pipelines to align to the human genome (reference build hs37d5) and identify germline variants and somatically acquired mutations, as described in the lead paper of the PCAWG Consortium^22^. We used SV breakpoint orientations as a measurement to classify deletions, inversions, duplications or complex rearrangements as described previously^23^. Complex rearrangements included chromothripsis^24^ and other alterations, which covered SV break-ends with concomitant deletions, inversions or duplications. SVs were further categorized into two subgroups based on the length of the events—SVs that were longer than 2 Mb in genomic length (long-range SVs) and shorter than 2 Mb in genomic length (short-range SVs). The majority of deletions, inversions and duplications could be categorized as short-range; however, complex events tended to be longer in length (Extended Data Fig. 2a). In this study, we focused on short-range SVs because long-range SVs could affect multiple boundaries due to the genomic length of the event. We identified SVs that affected the TAD boundaries (boundary affecting (BA)) as the ones that spanned the whole length of a boundary (around 75 kb). As a result, 5.0%, 8.5%, 12.8% and 19.9% of all deletions, inversions, duplications and complex events were called BA events, respectively (Fig. 1b). Compared with the expected number of boundary disruptions based on randomly shuffled boundaries, these ratios are strongly enriched in BA-duplications (*P* < 10^−4^, 1.43-fold enrichment). In contrast, we observed a depletion (0.87-fold enrichment, *P* = 0.052) in BA-deletions, whereas BA-inversions and BA-complex events occurred at expected levels (*P* > 0.05) compared with the shuffled TAD boundaries (Fig. 1c). Overall, these results suggest that deletions tended to occur within the same TAD, whereas duplications tended to span regions across different TADs.

In cancer cells, boundaries are affected to various degrees due to structural alterations, which suggests that some mechanistic differences could cause different SV types. Length distributions of the BA-SVs were uniformly distributed (Extended Data Fig. 2b). Most of the BA-SVs targeted a single boundary; 74% of BA-deletions, 65% of BA-inversions, 71% of BA-duplications and 64% of BA-complex events affected a single boundary per variant (Fig. 1d). The number of affected boundaries did not markedly change with the minimum length of the SVs (Fig. 1d, Extended Data Fig. 2c). The majority (98.4%) of the boundaries were affected in cancer genomes, although a few boundaries were located in the low-mappability regions of the genome. Interestingly, TAD boundaries are significantly less likely (*P* < 0.02) to be affected by known deletion and duplication polymorphisms derived from genomes of healthy human populations^25–27^ (Extended Data Fig. 2d). Genomic length of the germline alterations tends to be shorter compared with somatic alterations observed in tumors due to negative selection against large SVs in the germline^28^. Therefore, we selected germline and somatic deletions with a genomic length between 75 kb and 250 kb that occurred in all cancer samples (Fig. 1e). This filtering ensured that the selected somatic (median, 137 kb) or germline (median, 113 kb) deletions had the length potential to disrupt TAD boundaries. We observed that germline deletions that affected TAD boundaries were rare (less than 0.1%; 6 affected out of total 924 deletions) compared with somatic deletions (4.1%), even in cases in which similar genomic ranges and less than 1% of the total boundaries were affected by germline events, suggesting that germline variations in TAD boundaries may not be as well tolerated as similar somatic alterations.

### Chromatin folding disruptions are specific to histological subtypes

**Fig. 2:**
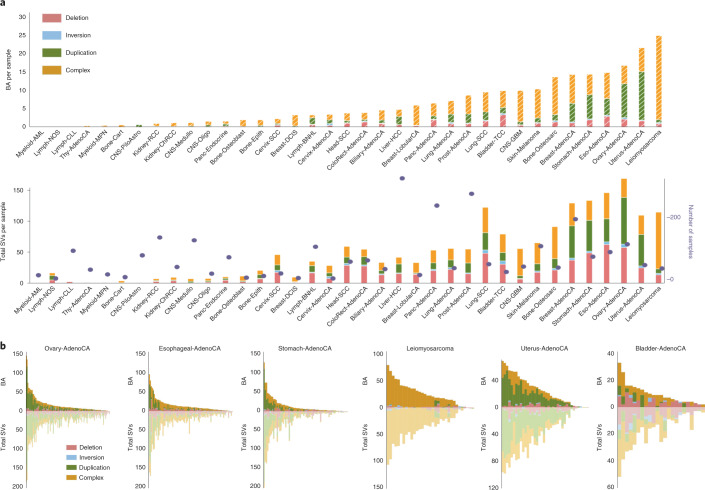
Chromatin folding disruptions are specific to histological subtypes. **a**, Top, the distribution of the average number of BA-SVs per sample for each histological type^22^. **Bottom**, The distribution of the average number of SVs observed in each histological type. Purple dots represent patient numbers for each histological type. Deletions, inversions, tandem duplications and complex rearrangements are shown in red, cyan, green and orange, respectively. **b**, Per-sample counts of BA-SV (top) and total SV (bottom) events for ovary, esophageal and gastric adenocarcinoma cohorts (left), and leiomyosarcoma, uterine adenocarcinoma and bladder adenocarcinoma cohorts (right). Deletions, inversions, tandem duplications and complex rearrangements are shown in red, cyan, green and orange, respectively. Each bar represents a sample and samples are sorted by the number of BA-SV events.

**Extended Data Fig. 3:**
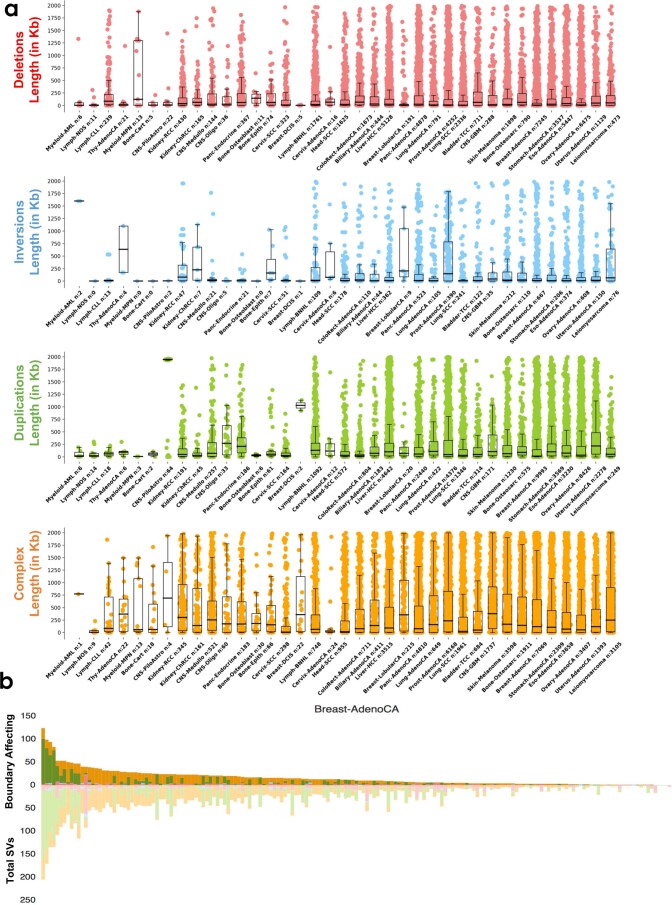
Histology-specific features of boundary-affecting structural variations. **a**, Box plots show the length (in Kb) distribution of short-range SVs (deletions: red, inversions: cyan, duplications: green) for each cancer histology subtypes^22^. The center line is the median; box limits are the upper and lower quantiles; whiskers represent 1.5x the interquartile range. Number of SVs are indicated by each histology name. **b**, Per sample counts of BA-SVs (top) and total SV (bottom) events for breast adenocarcinoma cohort. Deletion, inversions, tandem-duplications and complex rearrangements are represented with red, cyan, green and orange colors, respectively. Each bar represents a samples and samples are sorted by the number of BA-SV events.

**Extended Data Fig. 4:**
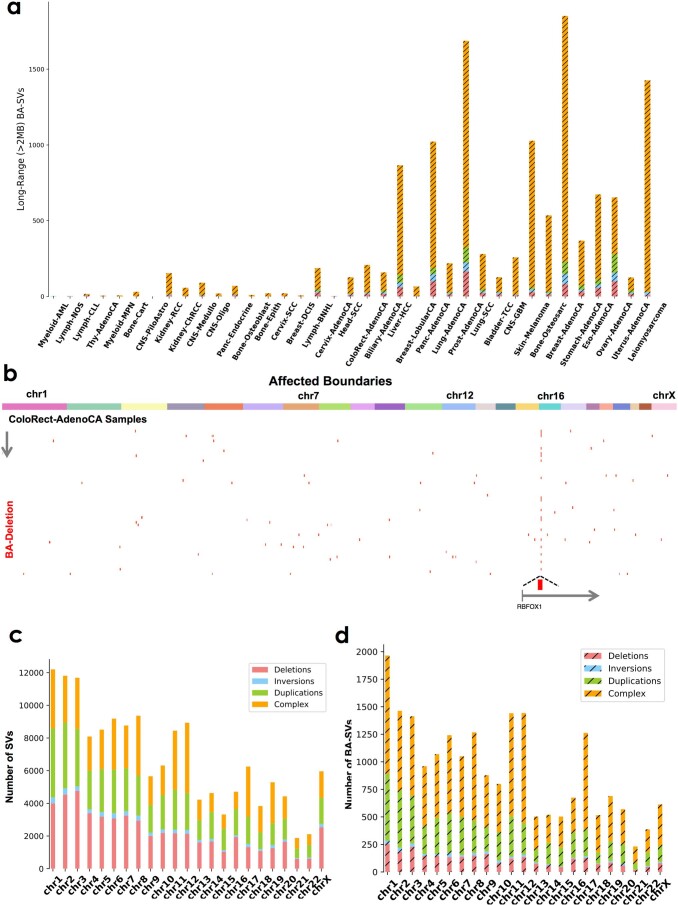
Further investigation of histology-specific features of boundary-affecting structural variations. **a**, Distribution of average long-range (length of SV>2 Mb) structural variations (deletion (dashed-red), inversion (dashed-cyan), duplication (dashed-green) and complex rearrangements (dashed-orange)) per sample for each cancer histology subtypes. **b**, A recurrently deleted TAD boundary in colorectal adenocarcinoma samples near to the *RBFOX1* gene. Colored bars on top depict chromosomal locations of the boundaries. Columns of the heatmap are TAD boundaries and rows represent each colorectal adenocarcinoma sample. TAD boundaries affected by BA-deletions are colored in red. Schematic below show the deleted boundary (red box) near to the *RBFOX1* gene. **c**, Distributions of total SV burden (deletions: red, inversions: cyan, duplications: green, complex: orange) across chromosomes. **d**, Distributions of boundary affecting SVs across chromosomes.

We next focused on the distributions of BA-SVs across 38 different histological cancer subtypes^22^. The number of BA-SVs generally followed the total number of SVs in a given cancer type. Our analysis revealed that, among all cancer types, leiomyosarcoma and uterus adenocarcinoma had higher numbers with—on average—25 and 22 BA-SVs per sample, respectively, compared with a median of around 7 BA-SVs per sample across all cancer samples (Fig. 2a, b). Ovarian, esophageal and breast adenocarcinomas also contained high numbers of BA-SVs with—on average—20, 19 and 18 BA-SVs per sample, respectively. On the other hand, hematopoietic cancers (myeloid-MDS or myeloid-AML) had the lowest BA-SV rates. Only glioblastoma samples (CNS-GBM) showed lower-than-expected BA-SVs (*P* < 10^−3^) across all cancer types. The median SV length of a given cancer type was not strongly correlated with the observed distributions (*r*^2^ = 0.03–0.45) (Extended Data Fig. 3a). The observed differences in BA-SV rates are likely driven by the differences in the burden and mechanisms of SVs across histological types. For instance, leiomyosarcoma and esophageal adenocarcinoma had a higher complex SV burden and, as a result, observed BA events were also mostly complex rearrangements (Fig. 2b), whereas ovary and stomach adenocarcinoma samples contained BA-duplications due to an overall higher duplication rate (Fig. 2b). Similarly, the total number of SVs in an individual tumor affects the observed BA-SVs in that sample (Fig. 2b, Extended Data Fig. 3b). Long-range BA-SVs had similar distributions across histological types. Again, leiomyosarcoma and breast adenocarcinoma contained a higher number of BA-SVs compared with other cancer types, whereas leukemia samples had no BA-SVs per sample (Extended Data Fig. 4a). Taken together, our findings show that the impact of BA-SVs is varied substantially across tumor types and these events were reflective of overall SV burden and type.

### Recurrently affected boundaries in specific cancer types

**Fig. 3:**
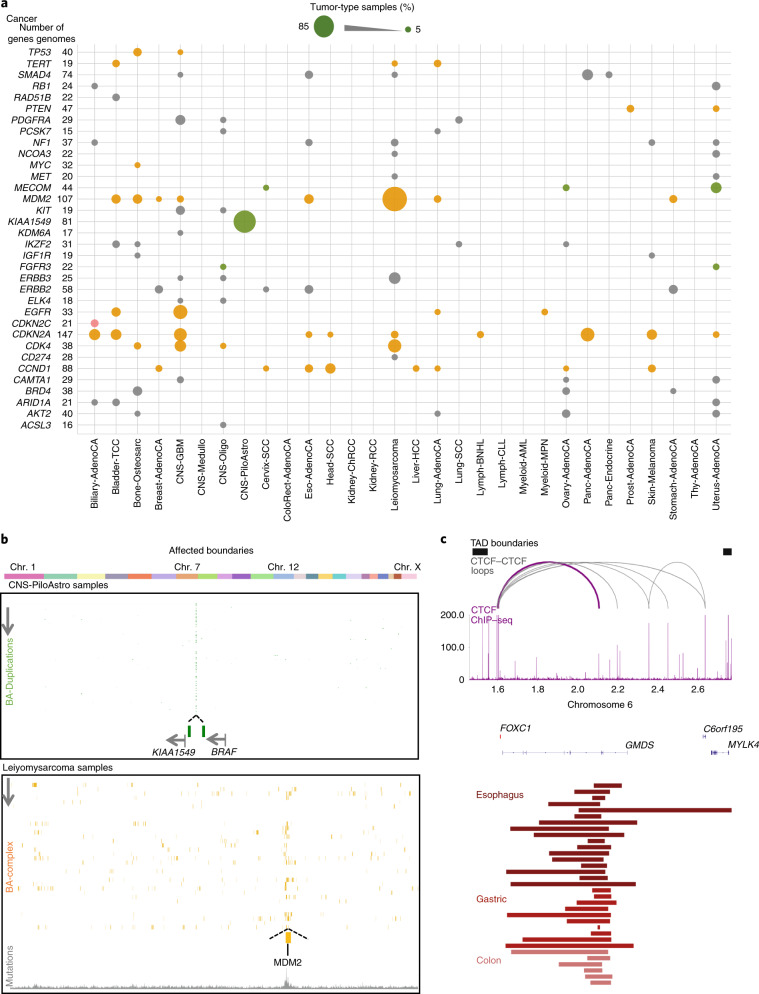
Recurrently affected boundaries in specific cancer types. **a**, Recurrently affected boundaries near known cancer driver genes (*y* axis) per histological type (*x* axis). The size of the circles indicates the portion of samples harboring a BA-SV event in a specific histological type. The color of the circles demonstrates the most common SV type (red, deletion; orange, complex; green, duplication; gray, different SV types were observed) in each histological type. **b**, Top, recurrently duplicated TAD boundaries in pilocytic astrocytoma. Columns of the heat map are TAD boundaries and rows represent each pilocytic astrocytoma sample. TAD boundaries affected by BA-duplications are colored in green. The schematic at the bottom shows the duplicated boundaries (green boxes) between *KIAA1549* and *BRAF*. Bottom, distribution of BA-complex rearrangement (orange) events across chromosomes in each leiomyosarcoma sample. Heat maps show affected TAD boundaries in each leiomyosarcoma sample. The line plot at the bottom shows normalized mutation count. **c**, A potentially affected CTCF–CTCF chromatin loop in esophageal, gastric and colon adenocarcinoma near *FOXC1*. Black boxes show TAD boundaries, arcs represent common CTCF–CTCF loops observed in three different cell types (gray). The signal from CTCF chromatin immunoprecipitation followed by sequencing (ChIP–seq) (from the NHEK cell line) analysis is represented by a purple histogram. Red vertical bars indicate deletions in individual samples of esophageal, gastric and colon adenocarcinoma.

**Extended Data Fig. 5:**
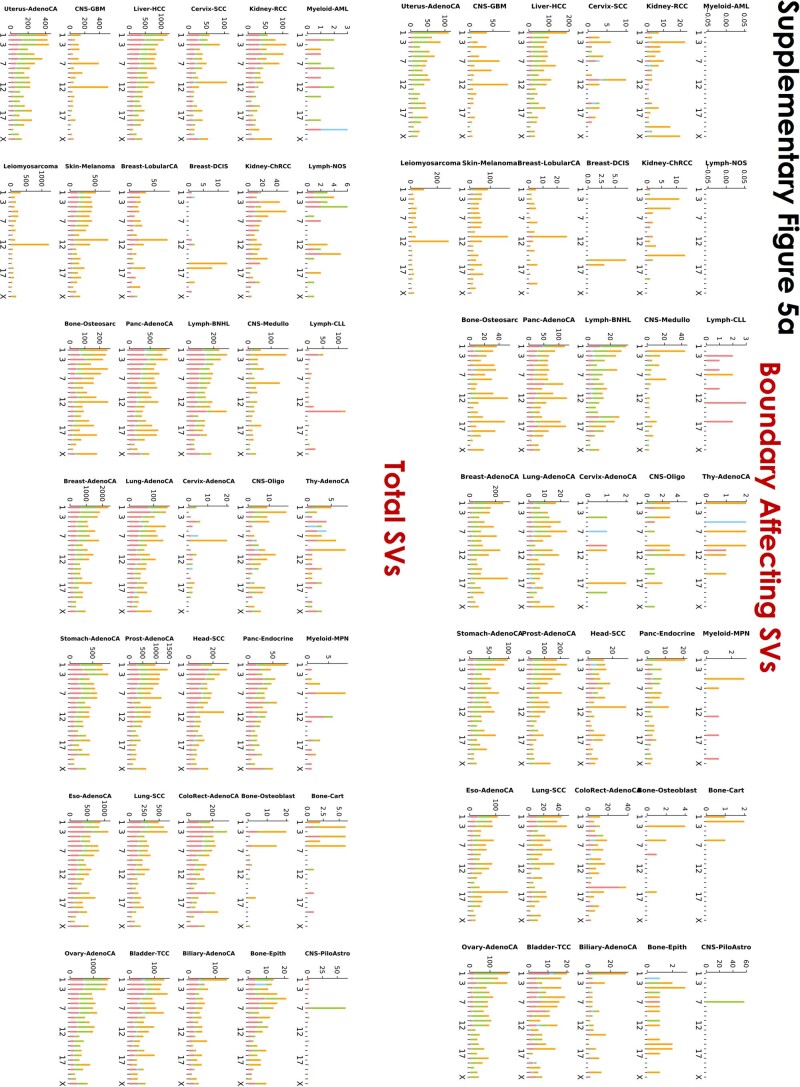
Distribution of structural variation burden in different cancer histology subtypes. **a**, Distribution of boundary-affecting (top) and total (bottom) SVs (deletions: red, inversions: cyan, duplications: green, complex: orange) across chromosomes in each cancer histology subtypes^22^.

**Extended Data Fig. 6:**
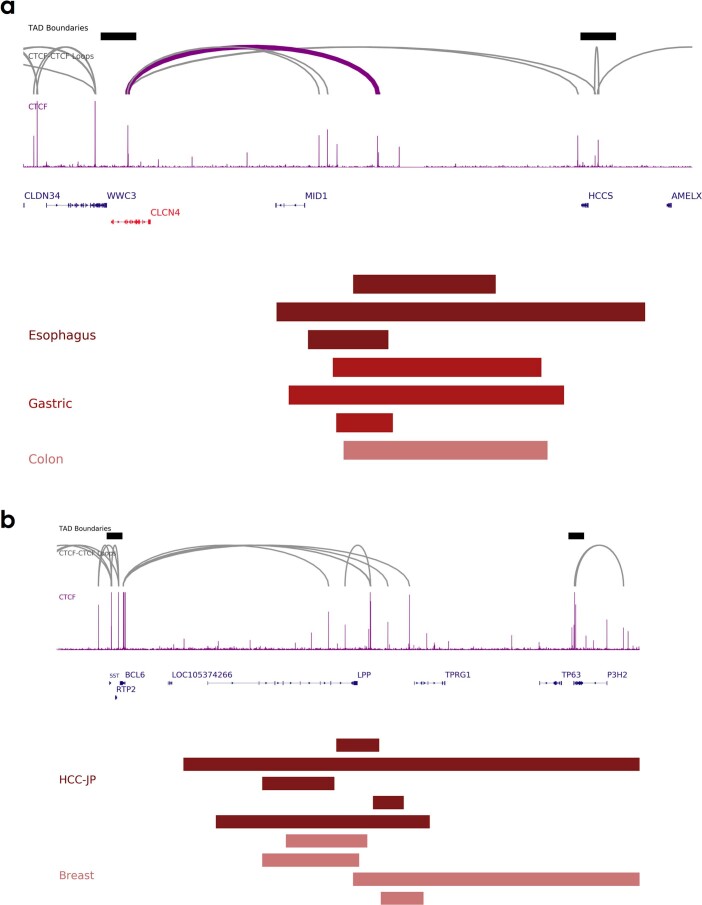
Examples of genomic alterations that potentially affect CTCF-CTCF chromatin folding loops. **a-b**, Potentially affected insulated neighborhoods **a**, in esophageal, gastric and colon adenocarcinoma samples near to the *CLCN4* gene and **b**, in liver-HCC and breast cancers near to *BCL6* gene. Black boxes show TAD boundaries, arcs represent CTCF ChIA-PET loops observed in three different cell types (gray). CTCF ChIP-Seq (from NHEK cell line) signal is represented by purple histogram. Red vertical bars depict deletions in individual samples.

Next, we sought to identify the affected boundaries near known driver genes in the COSMIC cancer gene census^29^. We noted that many of the boundaries of cancer driver genes were altered in specific histological subtypes (Fig. 3a, Supplementary Table 2). Of those recurrently affected boundaries, two adjacent boundaries between *KIAA1549* and *BRAF* were prone to BA-duplications specifically in samples of pilocytic astrocytoma (Fig. 3b). This region has previously been implicated in pilocytic astrocytoma, producing an oncogenic fusion between the aforementioned genes^30^. In addition, boundaries near the *MDM2* locus were most affected in leiomyosarcoma (Fig. 3b), likely due to neochromosome formations that included the *MDM2* and *CDK4* genes^31^. We also observed a higher mutational load specifically on chromosome 12 in leiomyosarcoma samples (Fig. 3b). Another recurrent BA-SV event was the high number of BA-deletions around *RBFOX1* in colorectal adenocarcinoma samples (Extended Data Fig. 4b). We surveyed the BA-SV distributions on individual chromosomes and observed a positive correlation with the number of boundaries (*r*^2^ = 0.68–0.92) and gene density (*r*^2^ = 0.7–0.85) on a given chromosome (Extended Data Fig. 4c,d). Notably, distributions of BA-SVs per chromosome were generally specific to the histology subtype; for example, chromosome 17 was affected predominantly by BA-complex events in breast and esophageal adenocarcinoma samples (Extended Data Fig. 5a,b). These findings emphasize the cancer specificity of BA-SVs, in which active mechanisms lead to the overall SV burden and type in different tumor types yield potential changes in TAD structures, especially around cancer driver genes. We next examined SVs that occurred within TADs, which potentially resulted in the disruption of CTCF–CTCF chromatin loops^32^. We identified a number of chromatin loops that were potentially disrupted in various cancer types (Supplementary Table 3). For instance, a CTCF site near *FOXC1* overlaps with recurrent deletions in esophageal, gastric and colon adenocarcinomas (Fig. 3c). Other potentially altered loops include a CTCF site near *BCL6* in hepatocellular carcinoma and breast adenocarcinoma, and *CLCN4* in colorectal adenocarcinomas (Extended Data Fig. 6a,b). Therefore, chromatin folding perturbations can occur at various scales, include TADs and CTCF–CTCF chromatin loops in cancer genomes and recurrently altered boundaries are generally cancer-type specific.

### Most domain disruptions do not result in marked gene-expression changes

**Fig. 4:**
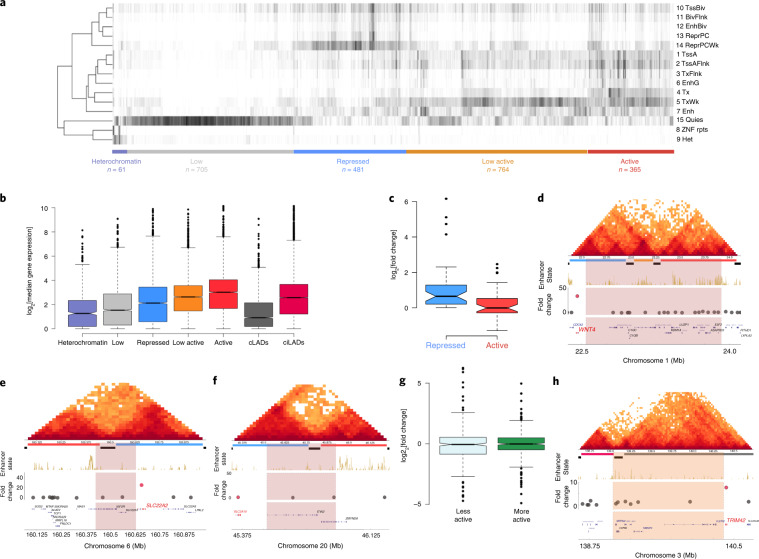
Most domain disruptions do not result in marked gene-expression changes. **a**, Classification of TADs based on chromatin state coverage. The heat map shows domain-length normalized coverage of each chromatin state (rows) in each domain (columns). Domains are classified into five groups according to chromatin state combinations: heterochromatin (purple), low/quiescent (gray), repressed (blue), low active (orange) and active (red). **b**, Median expression levels (log_2_) in all PCAWG samples are shown for genes located in each TAD, constitutive LADs (cLADs) and inter-LADs (ciLADs). The number of genes in each annotation group: heterochromatin, 624; low, 2,874; repressed, 3,690; low active, 4,319; active: 4,578; constitutive LAD, 2,384; constitutive inter-LAD, 14,430. In these and all other box plots, the center line is the median; box limits are the upper and lower quantiles; whiskers represent 1.5× the interquartile range. **c**, The log_2_-transformed fold change in expression is shown for genes that are nearest to BA-deletion break-ends between repressed and active TADs (*n* = 43). **d**–**f**, Examples of BA-SV-harboring samples. Triangle heat maps represent chromatin contact frequency (log_2_) in NHEK cells. BA-SV regions are shaded. Colored tiles represent domains and black bars denote TAD boundaries. Roadmap Epigenome enhancer-state frequencies are shown as a yellow histogram. Dots show fold changes in expression in a lymphoma sample harboring a BA-deletion near *WNT4* (**d**), a breast adenocarcinoma sample harboring a BA-deletion near *SLC22A2* (**e**) and an ovarian adenocarcinoma sample harboring a BA-deletion near *SLC2A10* (**f**). **g**, The log_2_-transformed fold change is shown for genes that are nearest to BA-deletion break-ends (*n* = 341). Transcriptionally less and more active refer to the ordering of domain annotations in **a** (that is, a low domain is considered less active than a low-active domain). **h**, Gene expression fold change in a melanoma sample harboring a complex SV between a LAD and an inter-LAD near *TRIM42*.

**Extended Data Fig. 7:**
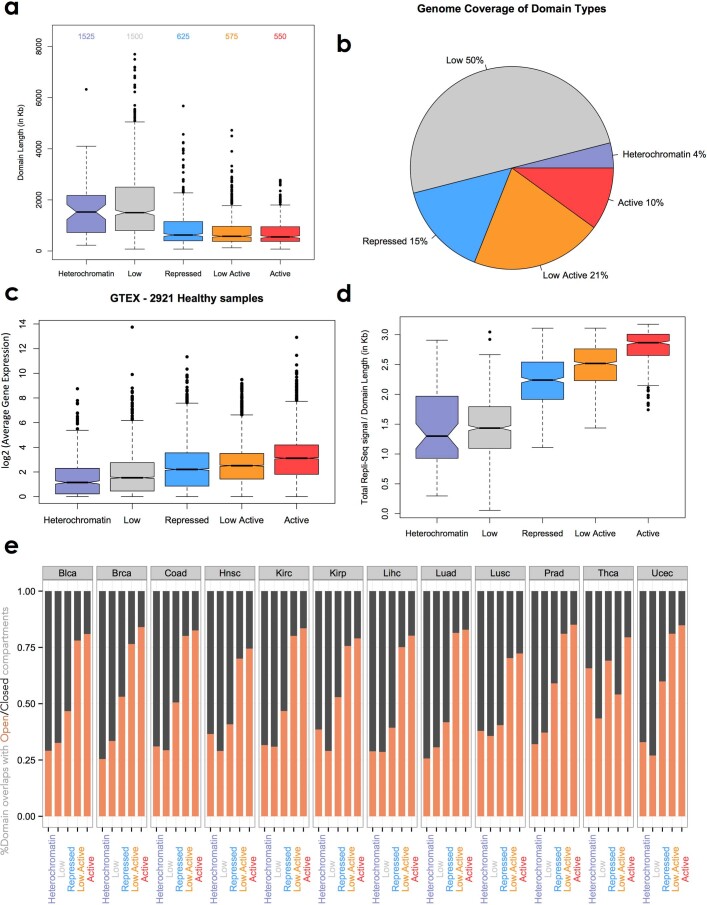
Classification of TADs based on the epigenetic landscape. **a**, Box plots show length distributions of different TAD annotations. Heterochromatin: 61; Low: 705; Repressed: 481; Low-Active: 764; Active: 365. In these and all other boxplots in subsequent figures, the center line is the median; box limits are the upper and lower quantiles; whiskers represent 1.5x the interquartile range. **b**, Pie chart represents percent of mappable genome covered by each TAD annotation. **c**, Box plots represent median expression level (RPKM) for a gene residing in a given TAD annotation for GTEX consortia dataset. Number of genes in each annotation group: heterochromatin: 624; low: 2874; repressed: 3690; low-active: 4319; active: 4578. **d**, Box plots represent replication timing (Repli-Seq) values divided by domain length (in Kb) for each TAD annotations. Heterochromatin: 61; Low: 705; Repressed: 481; Low-Active: 764; Active: 365. **e**, Bar plots show percent of a TAD annotation covered by open (orange) or closed (black) chromatin domains calls from a previous study^37^ across different TCGA cancer types.

**Extended Data Fig. 8:**
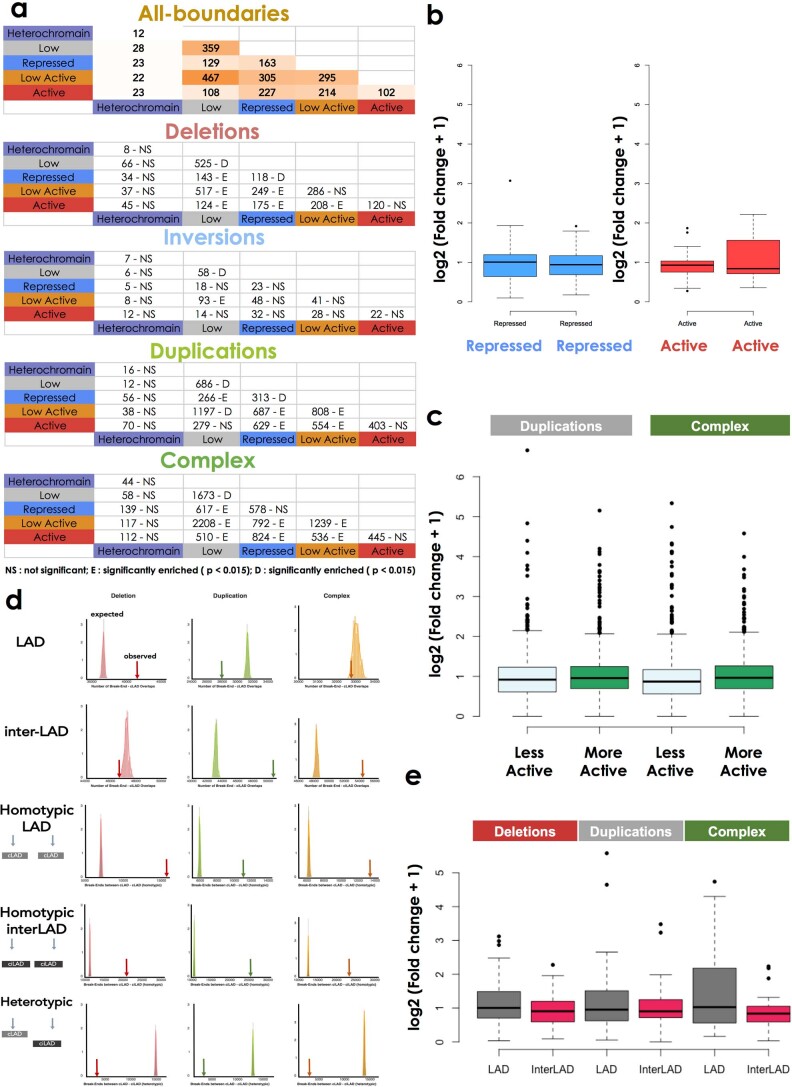
The majority of the domain disruptions do not result in drastic gene expression changes. **a**, Occurrence of different SV types between domain types. Significance of the observed numbers calculated based on the expected distribution which is based on randomly shuffled boundary data, cumulative distribution of expected overlaps, z-scores were calculated based on observed number and obtained distribution from this bootstrapping exercise A two-tailed unpaired Student’s t-test was used to calculate p-values. Significantly enriched (E) or depleted (D) numbers are denoted next to the numbers. **b**, Box plots show log2 fold-change for the genes nearest to BA-deletions between repressed-repressed (n: 19; blue; left) or active-active (n: 36; red; right) domains. In these and all other boxplots in subsequent figures, the center line is the median; box limits are the upper and lower quantiles; whiskers represent 1.5x the interquartile range. **c**, Box plots show log2 fold-change for the genes nearest to BA-duplication (n: 1008) and BA-complex (n: 617) break-ends on different domain types. Here ‘less’ or ‘more’ transcriptionally active refers to the ordering of domain annotations in Fig. 4a (that is a low domain is considered less compared to a repressed domain). Fold change was calculated based on the gene’s expression in the sample harboring the BA-SV compared to the rest of the samples in the same cancer type. **d**, Observed (arrows) and expected distribution (histograms) of SVs between constitutive LADs and interLADs. The expected distribution is based on randomly shuffled LAD and interLADs. **e**, Box plots show log2 fold-change for the genes nearest to deletion (n: 50), duplication (n: 66) and complex (n: 39) SVs between constitutive LAD and interLADs.

To ascribe potential functional effects of BA-SVs on chromatin domains, we annotated the TADs by profiling the context of aggregate chromatin states within each TAD. We used a probabilistic approach that calculated the occurrence of chromatin states in cell types recorded in the Roadmap Epigenome data. Coverage of 15 chromatin state enrichments in each domain was calculated and normalized to the length of the domain. The obtained matrix was grouped using the *k*-means clustering approach and five distinct groups of TADs were identified similar to a previous classification of chromatin domains^17,19,33^. These groups comprised heterochromatin (61), low/quiescent (705), repressed (481), low-active (764) and active (365) domains (Fig. 4a, Supplementary Table 4). In addition, we used constitutive LADs^34^ identified in three different human cell types to profile the outcomes of the SVs that occurred between LADs and inter-LADs. We evaluated the annotation results by profiling the distributions of domain sizes. Repressed domains were larger in size and covered the majority of the genome compared with active domains, in agreement with previous TAD annotations^19,35^ (Extended Data Fig. 7a,b). The median expression of genes within each domain was calculated for 2,921 cancer-free samples from 45 different tissues (GTEx consortium)^36^ as well as for samples from 998 patients with cancer from ICGC expression datasets. Analysis of expression levels confirmed that genes within repressed domains or LADs had significantly lower expression patterns than genes within active domains or inter-LADs (*P* < 2.2 × 10^−16^) (Fig. 4b, Extended Data Fig. 7c). Furthermore, distributions of replication timing for various cell types and open/closed chromatin compartment calls from TCGA data^37^ corroborated the data of the annotated domains (Extended Data Fig. 7d,e). Utilizing our domain annotations, we checked the distributions of flanking domains for BA-deletion, BA-inversion, BA-duplication or BA-complex events. The majority of the BA-SVs affected the same flanking domain types, such as boundaries that separated low and low domains or low-active and low-active domains (Extended Data Fig. 8a). However, BA-SVs between different domain types occurred significantly more frequently than the expected rate, which suggests that BA-SVs have a potential role in gene-expression changes (Extended Data Fig. 8a). Therefore, we compared expression values of the genes that reside on each side of the SVs.

We initially focused on BA-deletions between repressed and active domains, as previous studies showed that fused repressed–active domains could lead to an upregulation of nearby genes^38,39^. Indeed, genes located on the repressed side of deletions were significantly upregulated (*P* < 0.001, Supplementary Table 5) in samples with deletions compared with the rest of the samples in the same histological subtype (Fig. 4c), whereas the same effect was not observed for BA-deletions between repressed–repressed or active–active domains (Extended Data Fig. 8b). For example, a BA-deletion in a malignant lymphoma sample was associated with a 37-fold increase in the expression level of *WNT4* compared with the rest of samples from patients with lymphoma (Fig. 4d). Similarly, a BA-deletion in the genome of a patient with breast adenocarcinoma correlated with 26-fold overexpression of *SLC22A2* compared with the rest of the patients with breast cancer (Fig. 4e). However, this correlation of gene expression with BA-deletions between active and repressed domains was not universal. The fold change in expression of *SLC2A10* was 1.10 in a uterus adenocarcinoma sample with a BA-deletion compared with the rest of uterus tumor samples (Fig. 4f). Therefore, not every BA-deletion correlated with a marked change in gene expression; in fact, only 25% of BA-deletions between repressed and active domains coincided with twofold changes in gene expression (Supplementary Table 5). To use a higher number of events, we next extended our analysis to all BA-deletions that occurred between different domain types. We classified domains as ‘more’ or ‘less’ transcriptionally active based on the annotations of domains (the ordering of domain types is described in Fig. 4a). This analysis resulted in a non-significant (*P* > 0.05) difference between genes that were located on more or less transcriptionally active domains after BA-deletions (Fig. 4g); and 14% of all BA-deletions coincided with a twofold change (Supplementary Table 5). We observed a similar non-significant difference for BA-duplications and BA-complex events (Extended Data Fig. 8c, Supplementary Tables 6,7).

Next, we compared the events between LADs and inter-LADs to profile whether alterations in the lamin organization could contribute to gene expression in tumor samples. We observed that deletions significantly occurred in LADs and duplications in inter-LADs, whereas SVs were less likely to occur between LADs and inter-LADs (Extended Data Fig. 8d). We noticed certain correlations between gene expression and events between LADs and inter-LADs—for example, a complex rearrangement in a melanoma sample coincided with a sevenfold upregulation of *TRIM42* (which resides in a LAD) compared with the rest of the patients with melanoma (Fig. 4h). Overall, however, we did not observe a significant change for deletion, duplication and complex events between LADs and inter-LADs (Extended Data Fig. 8e, Supplementary Tables 8–10). These observations suggest that gene regulation in cancer genomes is multifactorial, although disruptions in chromatin folding domains may contribute to expression levels in certain cases, the effects of disruption do not always coincide with the expression changes.

### Cell-type-specific alterations in chromatin folding patterns by different SV types

**Extended Data Fig. 9:**
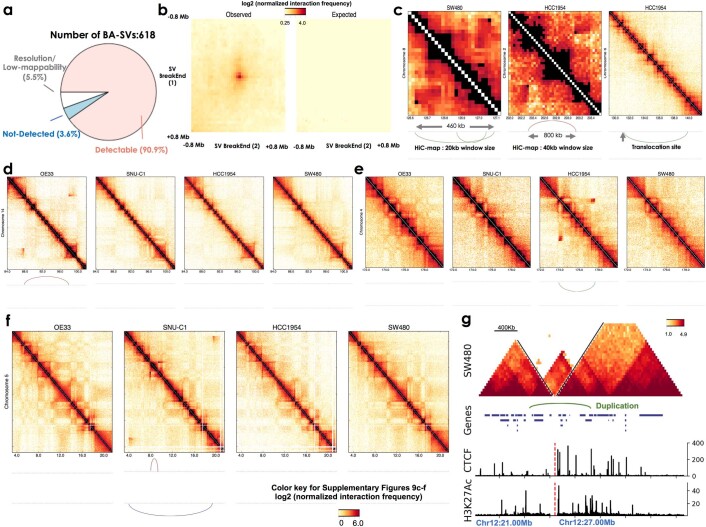
Cell-type specific alterations of chromatin folding patterns by different structural variation types. **a**, Pie chart represents the ratio of BA-SVs with detectable changes in Hi-C data from HCC1954, OE33, SNU-C1, SW480 cell lines. **b**, Average contact enrichment between break-ends of BA-SVs in cancerous and non-cancerous cell. Interactions between break-ends of BA-SVs longer than 1 Mb in length were included in this analysis. Breast epithelial cell line (HMEC) Hi-C data was used to represent non-cancerous cell interaction profile as the majority of BA-SVs in this analysis (56.3%) was detected in breast adenocarcinoma cell line (HCC1954). **c**, Examples of shortest BA-SVs with detectable changes in Hi-C maps and an SV with no detectable changes in Hi-C maps. Contact frequencies (log2) of each cell type, plotted with a 20KB (SW480) and 40Kb (HCC1954) window size. Arcs below represent SV breakpoint locations with rearrangements coded by color. Green: tandem duplication; red: deletion; cyan and purple: inversion. (Left) an 460Kb long duplication in SW480 cells; (middle) an 800 kb long deletion in HCC1954 cells; (right) a duplication overlapping with a translocation in HCC1954 cells resulted in no apparent contact map change. **d-f)** Represented regions for the effects of ‘simple’ genomic rearrangements on chromatin folding domains: **d**, A deletion on chromosome 4 in OE33 cells; **e**, A duplication on chromosome 14 in HCC1954 cells; **f**, A large inversion and a small deletion on chromosome 8 in SNU-C1 cells. **g**, A duplication (green arc) in SW480 cells results in a TAD-like formation on chromosome 4. Below histograms show CTCF and H3K27AC ChIP-Seq data from NHEK and SW480 cell lines, respectively. Red dashed line denotes the location of distinct genomic regions.

Next, to evaluate whether BA-SVs indeed altered chromatin folding patterns, we generated high-resolution Hi-C data for four cancer cell lines (SW480 and SNU-C1 for colorectal adenocarcinoma, HCC1954 for breast adenocarcinoma and OE33 for esophageal adenocarcinoma), which were previously profiled by WGS. For the majority of the BA-SVs detected by the WGS data (>90%), we were able to observe a change in the folding pattern in Hi-C contact maps of the respective cell line (Extended Data Fig. 9a). Break-ends of BA-SVs exhibited a strong contact frequency (14.6-fold) in cancer cells compared with non-cancerous cells (Extended Data Fig. 9b). The shortest BA-event with a detectable change in our Hi-C maps was a 460-kb long duplication in SW480 cells (Extended Data Fig. 9c). By contrast, we observed several discrepancies between SVs detected in WGS data and Hi-C maps. These SV break-ends tended to be located in repetitive regions of the genome or overlapped with inter-chromosomal translocations (Extended Data Fig. 9a,c). Our results demonstrate that BA-SVs detected using WGS data generally result in altered chromatin folding patterns in cancer cells.

**Fig. 5:**
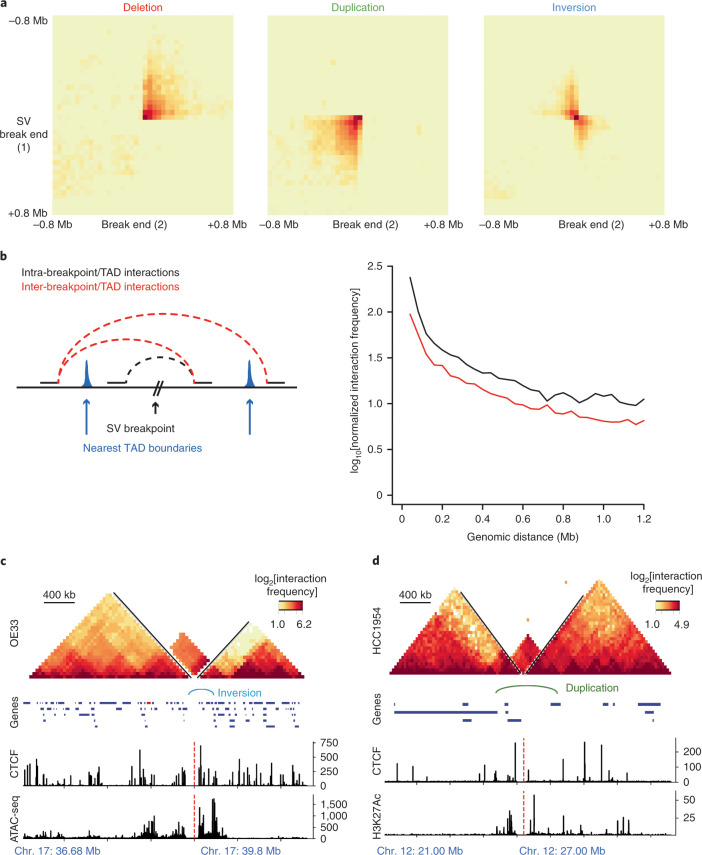
Cell-type-specific alterations in chromatin folding patterns associated with different SV types. **a**, Average contact enrichment between break-ends of BA-SV events in cancer cell lines. **b**, TAD-fusion analysis. The schematic shows the classification of interactions based on the nearest TAD boundary. Interactions between SV breakpoint and the nearest TAD boundary are classified as intra-TAD/SV (black dashed line) and interactions that are not constrained by the nearest boundary are classified as inter-TAD/SV (red dashed line). The decay plot shows how the interaction frequency changes as a function of genomic distance. **c**, **d**, Examples of neo-TAD formations by SVs in cancer genomes. Contact frequencies (log_2_) of each cell type, plotted with a 40-kb window size. Bottom arcs represent SV breakpoint locations with rearrangements coded by color. Green, tandem duplication; red, deletion; cyan and purple, inversion. Histograms show CTCF ChIP–seq data from NHEK, ATAC-seq data from OE33 and H3K27ac ChIP–seq data from HCC1954 cell lines. Red lines (dashed) denote the locations of distinct genomic regions. **c**, An inversion (blue arc) in OE33 cells led to a TAD fusion around *ERBB2*. **d**, A duplication (green arc) in HCC1954 cells resulted in a TAD-like formation on chromosome 4.

**Extended Data Fig. 10:**
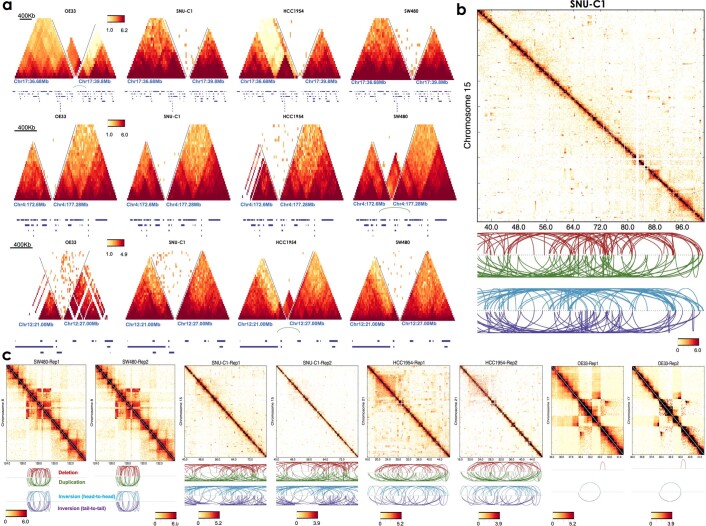
Specificity and reproducibility of chromatin organization alterations in cancer cell lines. **a**, Hi-C data around the neoTAD regions demonstrated in Fig. 5c and Supplementary Fig 10g in all cell lines. **b**, A smaller window of chromosome 15 represented in Fig. 5d which depicts a massive chromothripsis event covering all of the chromosome15 in SNU-C1 cell line. **c**, Biological reproducibility of SV’s effect on chromatin folding patterns represented for each Hi-C replicates of cell lines. Contact frequencies (log2) of each cell type, plotted with a 40Kb window size. Arcs below represent SV breakpoint locations with rearrangements coded by color. Green: tandem duplication; red: deletion; cyan and purple: inversion.

We subsequently studied how BA-deletions, BA-inversions, BA-duplications and BA-complex rearrangements change the contact maps and noticed distinct interaction patterns in chromatin contact maps for different BA-SVs (Fig. 5a, Extended Data Fig. 9d–f). This observation of specific changes in Hi-C maps due to different SV types is consistent with findings from a recent study^40^. Furthermore, it has also been suggested that SVs could lead to TAD fusions^40^ (also referred to as neo-TADs^3,4^); we therefore analyzed whether the BA-SVs observed in our cancer cell lines exhibited similar neo-TAD formation. We grouped bins on the basis of their location compared with the SV breakpoints and the nearest TAD boundary. If bins were between the SV breakpoints and the nearest TAD boundary, we classified these interactions as intra-TAD/SV and if bins were not constrained by the nearest boundary, we classified these interactions as inter-TAD/SV (Fig. 5b). Our analysis revealed that intra-TAD/SV interactions were stronger than the inter-TAD/SV interactions, when controlling for genomic distance effects, which suggests that the SVs can lead to cross-boundary interactions and potentially the formation of new chromatin folding domains based on the location of existing nearby TAD boundaries (Fig. 5b). For instance, an inversion in OE33 cells that encompassed *ERBB2* formed a neo-TAD on chromosome 17 (Fig. 5c), a duplication in HCC1954 cells on chromosome 4 (Fig. 5c) and a duplication near *KRAS* in SW480 cells (Extended Data Fig. 9g) resulted in a TAD-like configuration between previously disparate two TADs (Fig. 5c). These new TAD-like patterns could only be observed in cell lines that had the SV, suggesting that these folding patterns were the result of a specific alteration (Extended Data Fig. 10a). In all of these events, we observed that new interactions spanned the nearest boundary and formed ‘triangular shapes’ that were consistent with the TAD patterns observed in non-rearranged genomes. Therefore, BA-SVs have the potential to form new TAD structures in cancer cells that could reconfigure *cis*-regulatory interactions.

### Complex rearrangements markedly change chromatin folding maps in the cancer genomes

**Fig. 6:**
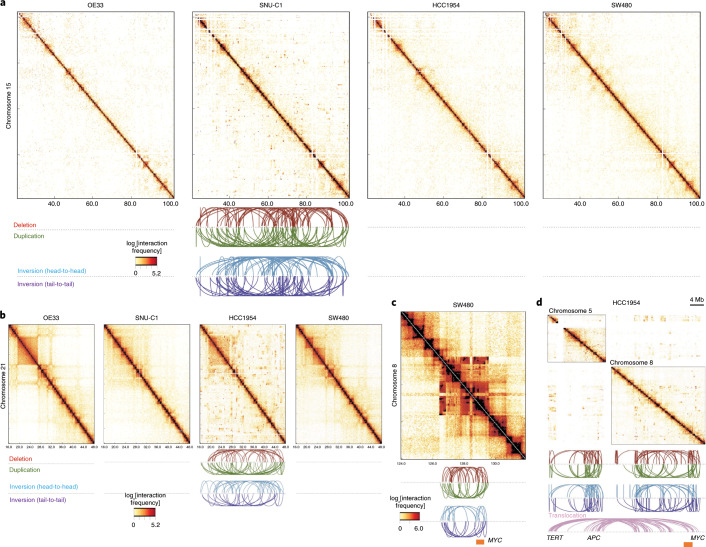
Complex rearrangements markedly change chromatin folding maps in the cancer genomes. **a**–**d**, The effects of complex rearrangements on chromatin folding domains. Contact frequencies (log_2_) of each cell type, plotted with a 40-kb window size. Bottom arcs represent SV breakpoint locations with rearrangements coded by color. Green, tandem duplication; red, deletion; cyan and purple, inversion. **a**, SNU-C1 cells harbor a chromothripsis event that affects chromosome 15. **b**, HCC1954 cells contain a complex rearrangement on chromosome 21. **c**, The *MYC* locus contains regional complex rearrangements in SW480 cells. **d**, A complex rearrangement that involves *TERT*, *APC* and *MYC* changes interactions between chromosome 5 and 8 in HCC1954 cells. Purple arcs represent inter-chromosomal translocations.

We noticed that complex rearrangements in which deletion, inversion or duplication break-ends overlapped resulted in marked changes in Hi-C maps. SNU-C1 cells contain a complex rearrangement (chromothripsis) across the entire chromosome 15, which was reported by WGS and spectral karyotyping^41^. This chromosome has 239 rearrangements in the SNU-C1 cells and we observed marked changes only in SNU-C1 Hi-C maps in which the differences in folding patterns overlapped with the identified SV break-ends (Fig. 6a, Extended Data Fig. 10b). Similarly, we noticed a chromothripsis-like event that covered chromosome 21 of HCC1954 cells in WGS data and, similarly, the Hi-C map of chromosome 21 in HCC1954 cells showed considerable changes (Fig. 6b). In addition to the complex rearrangements that covered whole chromosomes, we noticed regional complex rearrangements that had abnormal chromatin folding patterns. For example, the *MYC* locus in SW480 cells contains 135 rearrangements in a 4-Mb genomic window (Fig. 6c), whereas a larger complex event was observed in HCC1954 cells around the similar locus, which also involved two other cancer driver genes, *TERT* and *APC*, on chromosome 5 (Fig. 6d). We could detect the changes in biological Hi-C replicates, suggesting that these BA-SV effects are reproducible (Extended Data Fig. 10c). Given that complex rearrangements are the most frequent genomic alterations observed in the cancer genomes (Fig. 1b), studying the causes and consequences of these events using the chromatin conformation-based datasets would be critical for our understanding of the contribution of these events to the formation of cancer.

## Discussion

We explored the distributions of somatic SVs in a variety of tumor types and their potential roles in the disruption of chromatin folding and gene regulation. We found that certain boundaries are affected in a cancer-specific manner, which was likely due to the distribution of cancer-specific driver genes. Additionally, we observed a difference between the disruptions between different SV types; deletions tended to occur within TADs and LADs, whereas duplications tended to span TADs and generally occurred within inter-LAD regions. These results suggest that mechanistic differences may underlie the generation of different types of SV. For example, genome organization may influence partner selection during genomic rearrangements, as suggested by the distribution of different SV types in the genome to varying degrees. Disruption of folding domains could result in aberrant interactions between flanking domains and potentially contribute to the re-shaping of gene expression around the affected regions. Notably, we did not observe a strong association between global changes in gene expression after the disruption of each TAD, and only 14% of overall cases resulted in upregulation of more than twofold, which is consistent with the findings of recent studies^42,43^. These low expression changes may be reminiscent of mutations, in which there is a subset of chromatin-scale events that may be more likely to have functional effects (drivers) among a backdrop of considerable passenger events. Although we compared expression patterns of tumors in this study, cancer genomes may have other alterations that could affect the observed gene expression patterns, including copy-number alterations, dysregulation of transcription factors, chromatin regulators or *cis*-regulatory elements^44^. Therefore, the availability of histology-specific matched control samples coupled with WGS and chromatin organization datasets will augment our understanding of the functions of SV in genome folding and transcriptional dysregulation in cancers and contribute to our ability to discern signal from noise in appropriate contexts.

## Methods

### Hi-C data analysis

Chromatin conformation assay (Hi-C) data for cell lines of GM12878, HUVEC, IMR90, HMEC, NHEK and K562 were downloaded from GEO (GSE63525). Intra-chromosomal 25-kb-resolution raw observed, MAPQGE30-filtered values were normalized by dividing by the multiplication of Knight and Ruiz normalization scores for two contacting loci. We calculated the TAD signal by moving a window across the Hi-C matrix diagonal, the sum of the interaction for a given bin of up to 2-Mb flanking regions and log_2_ of the observed bin to the mean of interaction values within the given 2-Mb window. To identify TAD boundaries, we used an approach that is based on insulation score calculation^18^, and called TAD boundaries for each chromosome of each cell line with the following parameters: ‘-is 1000000 -ids 200000 -im mean -bmoe 1 -nt 0.1 --v’.

To calculate the significance of overlap between different TAD boundary calls, we converted the boundary regions into binary bins per genome to compare the overlap between previously published IMR90 TAD boundaries^4^ with our IMR90 boundary calls. We performed logical AND operation, in which the region is counted as overlapping boundaries between two datasets if only two bins for the same genomic location of each condition are 1. We used bootstrapping to determine the distribution of the random overlap numbers between two calls, and calculated *P* values based on the observed number and distribution of the shuffled boundaries. Shuffled boundaries are generated by randomly assigning boundaries while keeping the number of boundaries per chromosome constant. Obtained shuffled boundaries were also converted to binary string and the same logical AND operation was applied. Shuffling was performed 10,000 times for a given boundary set. This procedure is applied in the rest of our study to generate shuffled boundaries. Next, we computed cumulative distribution of expected overlaps, *z*-scores were calculated based on the observed number and obtained distribution from bootstrapping. A two-tailed unpaired Student’s *t*-test was used to calculate *P* values.

Common TAD boundaries were identified for boundaries of all five cell-types (GM12878, HUVEC, IMR90, HMEC and NHEK) that occurred within two Hi-C bins or 50 kb in genomic range. The same bootstrapping method (described above) was applied to calculate the significance of the overlap between common boundaries with TAD boundaries from the cancer cell lines K562 and MCF7.

To cluster individual TADs (defined as genomic regions between two adjacent common boundaries) based on epigenetic modifications, we used a comprehensive epigenome-profiling dataset from various human cell types. To this end, we used an entropy-based approach (epilogos) to calculate the occurrence of each chromatin state enrichment for a given genomic region across all cell types profiled by Roadmap Epigenome Consortia (http://compbio.mit.edu/epilogos/). We calculated the ratio of a TAD genomic space covered by each chromatin state, divided by the length of the TAD, and generated a normalized matrix in which columns are TADs and rows are each chromatin state, which have been extensively studied by the Roadmap Epigenome Consortia^21^. We applied hierarchical clustering to rows to identify similar chromatin states and *k*-means clustering to columns to group TADs that contain similar epigenetic modifications. We performed *k*-means clustering with *k* = 2–8 clusters and decided on *k* = 5 clusters as previous chromatin studies^17,19^ have used 5 distinct epigenetically modified chromosomal domains and *k* = 5 corresponded to better visually discernible domains. To determine how our TAD clustering correlate with gene expression in cancer-free and cancerous tissues, we downloaded normalized gene expression values for 2,663 different cancer-free samples from the GTEx Portal^36^ (v.1.6) and used normalized gene-expression values for ICGC cancer samples. We plotted the median expression of the genes in GTEx and ICGC samples, located in each domain type. Expression differences between heterochromatin and repressed domain expression with active domain expression were tested with one-tailed Mann–Whitney *U*-test. We also calculated the total number of genomic regions covered by each domain type. Finally, identified open and closed chromatin compartments (at a 100-kb resolution) in cancer samples using DNA methylation levels were identified as described previously^37^. We determined the percentage of our domain calls covered with open and closed chromatin calls from available cancer types.

We used HiCPlotter^45^ to plot Hi-C data with different features, TAD boundaries or gene-expression fold changes after deletion between repressed and active domains.

### ENCODE and Roadmap data

ENCODE replication timing data were downloaded from the UCSC Genome Browser ENCODE portal for the following cell types: BJ, GM06990, GM12801, GM12812, GM12813, GM12878, HeLa-S3, HepG2, HUVEC, IMR-90, K-562, MCF-7, NHEK and SK-N-SH. Replication timing values for smoothed wavelength transformed data were binned into 25-kb windows across the genome to discretize the data. Averages of the values in each bin across all cell types were calculated and used as average replication timing throughout the study.

We downloaded CTCF binding sites and DNase I hypersensitivity for five cell types (GM12878, HUVEC, IMR90, HMEC, NHEK) from the UCSC Genome Browser ENCODE portal. In addition, H3K9me3 and input DNA ChIP–seq alignment files (.bam) for each cell type were also downloaded. We randomly selected the same number of alignment reads for H3K9me3 and input DNA from .bam files and calculated log_2_-transformed enrichment levels of H3K9me3 over input DNA.

We downloaded all available CTCF peak-calling results and DNase I hypersensitivity regions from the UCSC Genome Browser ENCODE portal from 80 and 115 different cell lines, respectively (Supplementary Table 11). Occurrences of CTCF-binding and DNase I hypersensitivity sites per 25-kb window across the genome were calculated for all downloaded cell types and used to calculate TAD boundary and shuffled boundary enrichments.

### Structural alterations

Somatic and germline variant calls, mutational signatures, subclonal reconstructions, transcript abundance, splice calls and other core data generated by the ICGC/TCGA PCAWG Consortium are described by the lead paper^22^ of the PCAWG Consortium and available for download at https://dcc.icgc.org/releases/PCAWG. Additional information on accessing the data, including raw read files, can be found at https://docs.icgc.org/pcawg/data/. In accordance with the data access policies of the ICGC and TCGA projects, most molecular, clinical and specimen data are in an open tier that does not require access approval. To access potentially identifying information, such as germline alleles and underlying sequencing data, researchers will need to apply to the TCGA Data Access Committee (DAC) via dbGaP (https://dbgap.ncbi.nlm.nih.gov/aa/wga.cgi?page=login) for access to the TCGA portion of the dataset, and to the ICGC Data Access Compliance Office (DACO; http://icgc.org/daco) for the ICGC portion. In addition, to access somatic SNVs derived from TCGA donors, researchers will need to obtain dbGaP authorization.

We obtained the consensus SV calls and annotations of each variation (deletions, inversions, duplications and complex rearrangements), which can be found at Synapse (https://www.synapse.org/) with accession number syn7596712. The SV classification algorithm is comprehensively defined in another study^23^. The code for the classification algorithm is available on GitHub (https://github.com/cancerit/ClusterSV/). In brief, this algorithm clusters individual SV junctions into SV events that may involve multiple junctions. The single junction events were interpreted, as the ‘basic’ SV types (deletion, tandem duplication, translocation and inversions). However, in many cases events involving multiple SV junctions were detected. The SV events that involved many SV junctions could not be classified into any simple SV types. Therefore, these SV events were classified as complex. We specifically focused on the events that occurred within a chromosome in this study; we therefore did not use the translocation event calls between different chromosomes. To understand the effects of SVs, we first grouped the deletions, inversions or duplications on the basis of the length of the SVs.

Short-range SVs were identified as events with a length of less than 2 Mb and we mainly focused on these events in this study. BA-SVs were identified as SVs that spanned the whole length of a TAD boundary, the rest of the SVs were classified as ‘within TAD’ in Fig. 1b. To determine the distribution of random BA-SV events, we used the same bootstrapping method mentioned above, mainly generated random boundary events 10,000 times and calculated random BA-SV event distributions. The *z*-scores and *P* values were calculated on the basis of the observed number and distribution obtained from bootstrapping. In this study, we analyzed each event separately for deletion, duplication and inversion calls, albeit in a given sample these events might occur concurrently.

Long-range SVs were identified as events with a length of more than 2 Mb and we mentioned the results obtained with long-range SVs in the main text, as appropriate.

To understand the germline BA-SV occurrences, we downloaded structural alteration calls from three different studies: deletion events (total of 8,941) from WGS data of the 1000 Genomes project^26^; deletions (total of 7,511) and duplications (total of 7,501) from WGS data from 236 individuals representing 125 human populations^27^; and from a comprehensive review of deletions (total of 11,530) and duplications (total of 1,170) events from 23 different studies including 2,647 different individuals^25^. We noticed that the number of BA-SVs present in germline deletions and duplications was low and these events happened less than expected by chance, which was estimated using a bootstrapping method.

We next profiled short-range SVs and BA-SVs for each of the cancer studies in our ICGC dataset. To calculate the average number of SVs or BA-SVs per sample for each of the cancer studies, we divided the sum of all observed short-range SVs or BA-SVs in a given cancer type by the total number of samples in that cancer study. Observed SVs and BA-SVs across cancer studies were plotted as stacked bar charts representing deletions, inversions and duplications.

To identify the recurrently affected boundaries in each cancer study, we generated a matrix in which each column represented a sample in the cancer study and rows represented the TAD boundaries. A binary score was assigned to each row (a TAD boundary) that indicated whether that boundary was affected by BA-SV(s) in a given sample. Boundaries that were affected in more than 10% of the samples in a cancer study, are reported as recurrently affected boundaries in Supplementary Table 2. The median length of SVs per cancer type was calculated for all observed short-range SVs in each cancer type and plotted with the standard deviation of lengths. Constitutive insulated neighborhoods were obtained from Supplementary Table 8 of a previous study^15^ and SVs that affected only one anchor (CTCF-binding site) of an insulated neighborhood were considered as loop-disrupting SVs.

We determined flanking domain annotations of BA-SVs, by identifying the type of the nearest domain for the break-ends of each BA-SV. This analysis resulted in a half-matrix that contained the observed frequencies of pair-wise flanking domain types. We plotted the observed values for BA-SV deletions, inversions, duplications or complex rearrangements separately. To understand the genomic distribution of domain neighborhoods, we counted the flanking domains of each TAD boundary.

To profile SVs between nuclear LADs and inter-LADs, we obtained HMM state calls from three different human cell types for constitutive LADs and constitutive inter-LADs^34^ from GSE22428. For a filter, we used LAD calls from an independent study^3^. Genomic coordinates were converted to the hg19 assembly with the UCSC liftover tool. To calculate the significance of the observed overlaps between different SV types and constitutive LAD and constitutive inter-LADs, we used the same bootstrapping method, in which break-ends of each SV type were randomly shuffled on the same chromosome 10,000 times and *z*-scores were calculated between observed and expected values.

We identified the nearest genes to the break-ends of BA-SVs as the nearest RefSeq genes that did not overlap with the break-ends. The RefSeq gene table was downloaded from the UCSC Genome Browser in May 2016. We called genes located upstream of the 5′ end of an SV upstream genes and genes located downstream of the 3′ end of an SV downstream genes for each BA-SV. Fold changes in expression for each of the upstream and downstream genes were calculated by dividing observed normalized RPKM values in the particular sample with BA-SVs, with average normalized RPKM values in the rest of the same cancer study samples. We filtered the genes with low expression values (<0.1 FPKM), as fold changes with those genes would be seemingly high for even small fluctuations. Copy-number variations could be another confounding factor for observed gene-expression fold changes. Therefore, we obtained consensus copy-number calls for the ICGC cohort based on consensus SV results. We removed cases in which copy numbers are more than four for either the upstream or the downstream genes. In addition, we removed genes that were distal to the break-ends by more than 1 Mb. Expression differences between genes that flanked different BA-SV break-ends were tested using one-tailed Mann–Whitney *U*-tests.

We used pyvcf (https://pyvcf.readthedocs.org) to load .vcf files and pybedtools^46^ to perform genomic-interval analyses.

### Cancer cell lines

The colon cancer cell lines (SW480, SNU-C1) and breast adenocarcinoma cancer cell line (HCC1954) were obtained from the American Type Culture Collection and the esophageal adenocarcinoma (OE33) cell line was obtained from Sigma-Aldrich. Stocks were stored in liquid nitrogen. These cell lines were authenticated by comparing SV results from previous WGS datasets from the same cancer lines.

### WGS data analysis of cancer cell lines

We obtained the WGS datasets of the SW480, SNU-C1 and OE33 cell lines from previous publications^41,47,48^. To identify consensus SVs for SW480 and OE33 cell lines, we ran DELLY^49^, Lumpy^50^ and BRASS^51^ algorithms. SV breaks-ends reported by two different callers were included in this analysis. For the SNU-C1 cell line, SV calls were obtained from Supplementary Table 2 of a previous publication^41^, genomic coordinates were converted to the hg19 assembly using the UCSC liftover tool. HCC1954 whole-genome data were previously analyzed by the ICGC Structural Variation subgroup and we used the consensus structural alterations for this cell line.

### Cancer cell line Hi-C assay and analysis

Hi-C was performed using the in situ Hi-C protocol as previously described^17^ using 2–5 million cells per experiment that were digested with the MboI restriction enzyme and analyzed in duplicate. Hi-C libraries were sequenced on a NextSeq 500 or a HiSeq 4000. Reads were aligned to the hg19 reference genome using BWA-MEM^52^ and PCR duplicates were removed using Picard. Hi-C interaction matrices were generated using in house pipelines, and matrices were normalized using the iterative correction method^53^. ATAC-seq data for the OE33 cell line were obtained from a previous study^54^ and H3K27ac ChIP–seq datasets for the HCC1954 and SW480 cell lines were obtained from Hon et al.^55^ and Rahnamoun et al.^56^, respectively.

To investigate the potential function of SVs in TAD fusions, we classified the interactions on the basis of the nearest TAD boundary. For each SV, the average interaction frequency was calculated within a 2-Mb region of the SV. This average frequency ratio was used to ‘scale’ the interactions to account for ploidy. This was done by taking the average interaction frequency over that region and dividing it by the genome-wide average (controlling for the distance between loci) over a window of identical size. Certain WGS-defined SVs do not appear to have a signal in the Hi-C data, possibly due to false-positive SV calls, and we excluded regions for which the scaling factor was less than 0.1 to remove potential false-positive calls. In addition, we truncated the default 2-Mb window if there was another SV to avoid biases introduced by complex variants.

### Reporting Summary

Further information on research design is available in the Life Sciences Reporting Summary linked to this article.

## Online content

Any methods, additional references, Nature Research reporting summaries, source data, extended data, supplementary information, acknowledgements, peer review information; details of author contributions and competing interests; and statements of data and code availability are available at 10.1038/s41588-019-0564-y.

## Supplementary information

Supplementary Information Supplementary Note

Reporting Summary

Supplementary Tables Supplementary Tables 1–11

## Data Availability

Aligned sequencing data, as well as somatic and germline variant calls from PCAWG tumors, including SNVs, indels, copy number alterations and SVs, are available for download at https://dcc.icgc.org/releases/PCAWG. Additional information on accessing the data, including raw read files, can be found at https://docs.icgc.org/pcawg/data/. In accordance with the data-access policies of the ICGC and TCGA projects, most molecular, clinical and specimen data are in an open tier that does not require access approval. To access potentially identifying information, such as germline alleles and underlying sequencing data, researchers will need to apply to the TCGA Data Access Committee (DAC) via dbGaP (https://dbgap.ncbi.nlm.nih.gov/aa/wga.cgi?page=login) for access to the TCGA portion of the dataset, and to the ICGC Data Access Compliance Office (DACO; http://icgc.org/daco) for the ICGC portion. In addition, to access somatic SNVs derived from TCGA donors, researchers will also need to obtain dbGaP authorization. We obtained the consensus SV calls and annotations of each variation (deletions, inversions, duplications and complex rearrangements), which can be found at Synapse (https://www.synapse.org/) with accession number syn7596712. Hi-C data have been deposited at GEO under accession code GSE116694.

## Code availability

The core computational pipelines used by the PCAWG Consortium for alignment, quality control and variant calling are available to the public at https://dockstore.org/search?search=pcawg under a GNU General Public License v.3.0, which allows for reuse and distribution.
